# Comparative time-dependent proteomics reveal the tolerance of cancer cells to magnetic iron oxide nanoparticles

**DOI:** 10.1093/rb/rbae065

**Published:** 2024-06-04

**Authors:** Yanqing Liu, Yuqing Meng, Yongping Zhu, Liwei Gu, Ang Ma, Rui Liu, Dandan Liu, Shengnan Shen, Shujie Zhang, Chengchao Xu, Junzhe Zhang, Jigang Wang

**Affiliations:** State Key Laboratory for Quality Ensurance and Sustainable Use of Dao-di Herbs, Artemisinin Research Center, Institute of Chinese Materia Medica, China Academy of Chinese Medical Sciences, Beijing 100700, China; State Key Laboratory for Quality Ensurance and Sustainable Use of Dao-di Herbs, Artemisinin Research Center, Institute of Chinese Materia Medica, China Academy of Chinese Medical Sciences, Beijing 100700, China; State Key Laboratory for Quality Ensurance and Sustainable Use of Dao-di Herbs, Artemisinin Research Center, Institute of Chinese Materia Medica, China Academy of Chinese Medical Sciences, Beijing 100700, China; State Key Laboratory for Quality Ensurance and Sustainable Use of Dao-di Herbs, Artemisinin Research Center, Institute of Chinese Materia Medica, China Academy of Chinese Medical Sciences, Beijing 100700, China; State Key Laboratory for Quality Ensurance and Sustainable Use of Dao-di Herbs, Artemisinin Research Center, Institute of Chinese Materia Medica, China Academy of Chinese Medical Sciences, Beijing 100700, China; College of Animal Science and Technology, Henan Agricultural University, Zhengzhou 450002, China; State Key Laboratory for Quality Ensurance and Sustainable Use of Dao-di Herbs, Artemisinin Research Center, Institute of Chinese Materia Medica, China Academy of Chinese Medical Sciences, Beijing 100700, China; State Key Laboratory for Quality Ensurance and Sustainable Use of Dao-di Herbs, Artemisinin Research Center, Institute of Chinese Materia Medica, China Academy of Chinese Medical Sciences, Beijing 100700, China; State Key Laboratory for Quality Ensurance and Sustainable Use of Dao-di Herbs, Artemisinin Research Center, Institute of Chinese Materia Medica, China Academy of Chinese Medical Sciences, Beijing 100700, China; State Key Laboratory for Quality Ensurance and Sustainable Use of Dao-di Herbs, Artemisinin Research Center, Institute of Chinese Materia Medica, China Academy of Chinese Medical Sciences, Beijing 100700, China; State Key Laboratory for Quality Ensurance and Sustainable Use of Dao-di Herbs, Artemisinin Research Center, Institute of Chinese Materia Medica, China Academy of Chinese Medical Sciences, Beijing 100700, China; State Key Laboratory for Quality Ensurance and Sustainable Use of Dao-di Herbs, Artemisinin Research Center, Institute of Chinese Materia Medica, China Academy of Chinese Medical Sciences, Beijing 100700, China; Department of Nephrology, Shenzhen key Laboratory of Kidney Diseases, and Shenzhen Clinical Research Centre for Geriatrics, Shenzhen People’s Hospital, The First Affiliated Hospital, Southern University of Science and Technology, Shenzhen 518020, China

**Keywords:** iron oxide nanoparticles, tumor therapy, biosafety evaluation, cell death

## Abstract

Cancer is one of the most challenging diseases in the world. Recently, iron oxide nanoparticles (IONPs) are emerging materials with rapid development and high application value, and have shown great potential on tumor therapy due to their unique magnetic and biocompatible properties. However, some data hint us that IONPs were toxic to normal cells and vital organs. Thus, more data on biosafety evaluation is urgently needed. In this study, we compared the effects of silicon-coated IONPs (Si-IONPs) on two cell types: the tumor cells (Hela) and the normal cells (HEK293T, as 293 T for short), compared differences of protein composition, allocation and physical characteristics between these two cells. The major findings of our study pointed out that 293 T cells death occurred more significant than that of Hela cells after Si-IONPs treatment, and the rate and content of endocytosis of Si-IONPs in 293 T cells was more prominent than in Hela cells. Our results also showed Si-IONPs significant promoted the production of reactive oxygen species and disturbed pathways related to oxidative stress, iron homeostasis, apoptosis and ferroptosis in both two types of cells, however, Hela cells recovered from these disturbances more easily than 293 T. In conclusion, compared with Hela cells, IONPs are more likely to induce 293 T cells death and Hela cells have their own unique mechanisms to defense invaders, reminding scientists that future in vivo and in vitro studies of nanoparticles need to be cautious, and more safety data are needed for further clinical treatment.

## Graphical Abstract

**Figure:**
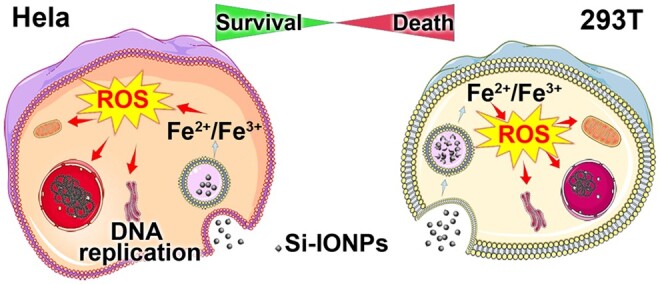

## Introduction

Cancer is one of the most challenging diseases in the world. Despite decades of continuous efforts to explore the mechanisms and treatments of tumor, no significant improvements have been observed in cancer therapy. The lack of tumor specificity and strong dose-related toxicity of chemotherapeutic agents are major obstacles in the current treatment of cancer. Nanomaterials have attracted the attention of many researchers because of their tumor-specific delivery potential while minimizing side effects, and nowadays nanomaterials play important and diverse roles in cancer treatment.

Iron oxide nanoparticles (IONPs) are nanomaterials with rapid development and high application value in recent years. Because of their unique magnetic properties, low toxicity, multifunctional capability, biocompatibility and biodegradability, IONPs have been widely used in many fields of biomedicine, such as immunoassay, magnetic resonance imaging (MRI), magnetic hyperthermia and drug delivery, and they are the only metal nanoparticles approved for clinical use by the US Food and Drug Administration (FDA) [1–3]. Recently, IONPs exhibit cytotoxicity in malignant cells which provides a new direction for antitumor therapy [4].

It was reported that IONPs could induce toxicity through causing oxidative stress, DNA strand breakage and induce caspase-3 activity in human breast cancer cells [5]. Moreover, the silica-coated IONPs (Si-IONPs) were considered to induce cancer cell death by restraining the intracellular membrane integrity and augmenting the calcium ions concentration [6]. IONPs also has been reported to induce cancer cell death via triggering ferroptosis which is a new type of iron-dependent programmed cell death that different from apoptosis, cell necrosis and autophagy [7–11]. IONPs endocytosed into cells can be degraded and release the ferrous or ferric ions leading to Fenton reaction, which in turn resulting in the generation of reactive oxygen species (ROS), lipid peroxidation and ferroptosis [12].

Although IONPs have shown great potential on tumor therapy due to their unique magnetic and biocompatible properties via causing oxidative stress and ferroptosis, the underlying mechanism of it still unclear and the data comparing the response of cancer cells and normal cells to IONPs is lacking, especially time-course monitoring. With the nowadays increasing exposure of IONPs in the biomedical field, there is also some data hint us that IONPs were toxic to normal cells and vital organs. Studies have shown that IONPs passively accumulated in spleen, lymph nodes and liver, which restricted its clinical use as MRI contrast agents [13, 14]. Thus, more data on biosafety evaluation of IONPs is urgently needed, and more research on the underlying mechanisms is required to optimize their therapeutic efficacy and safety before they can be widely used in clinical practice [15].

Mass spectrometry (MS)-based proteomics is a powerful tool which has been successfully used for identify and quantify proteins from single cells, different tissues or dynamic biological processes [16, 17]. To monitor the time-course cellular effects and analyze the different expressed proteins (DEPs) between cancer cells and normal cells after treatment with IONPs at the molecular level, proteomics is undoubtedly a powerful tool via providing qualitative and quantitative protein information [18]. A comparative proteomic technique might reveal considerable variation in protein fingerprints and trace the relationship between DEPs [19].

**Scheme 1.**
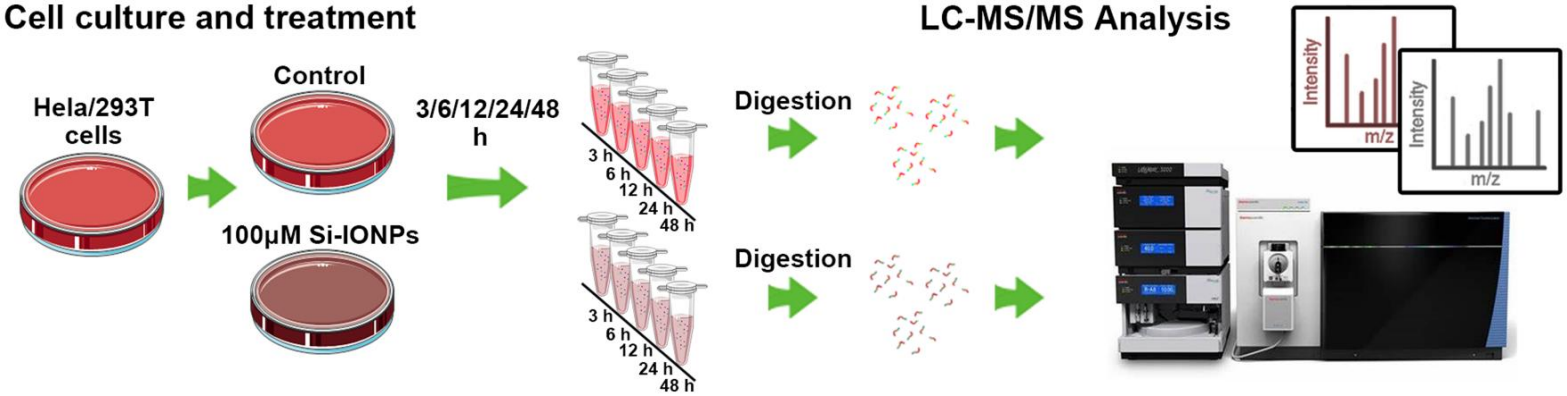
Illustration of this performed experiment.

In this study, we compared the time-course cellular effects of Si-IONPs on two types of cells, Hela cancer cells and HEK293T (293 T for short) normal cells and the dynamic physiological process during the two cells’ response to IONPs, as well as the differences and similarities of protein expression between these two cells after IONPs exposure (Scheme 1). Our data indicated that 293 T cells are more sensitive to Si-IONPs and prone to cell death compared with Hela cells. Transmission electron microscope (TEM) showed that Si-IONPs endocytosis rate of Hela cells was slower than 293 T. The results of MS-based proteomics showed significant changes in the signaling pathways related to oxidative stress, iron homeostasis, apoptosis, immune response and ferroptosis. Meanwhile, the in-depth studies on these pathways manifested that Hela cells have a stronger ability to adjust and adapt the stress caused by Si-IONPs and further resist cell death compared with 293 T cells. Our findings reveal that Si-IONPs are more toxic to normal cells than cancer cells, and cancer cells have their own unique mechanisms to defense invaders, reminding scientists that future *in vivo* and *in vitro* studies of nanoparticles need to be cautious, and more safety data are needed before further clinical trials.

## Materials and methods

### Si-IONPs nanoparticle preparation and characterization

The Si-IONPs were synthesized and characterized as previously described [20]. The detailed characterization results of the synthesized Si-IONPs were showed in supplementary file (Table S1).

### Cell culture

Immortalized human embryonic kidney cells and cancer cells (293 T/Hela) were used in this research. Cells were cultured in DMEM medium (Corning, USA) containing 10% fetal bovine serum and 1% penicillin/streptomycin (Corning, USA) at 37°C with 5% CO_2_ in humidified incubator. Si-IONPs (100 μM) was mixed into the culture medium directly and treated cells for different times (0/3/6/12/24/48 h), respectively.

### Cell viability detection

Cells were seeded in 96-well plate at a density of 5 × 10^3^ cells per well and cultured for 24 h. Then treated with different concentrations of Si-IONPs (0–300 μM), and each concentration contained four wells in parallel. Cell viability was evaluated via CCK-8 kit at 24/48/72 h post-treatment, respectively, and the absorbance at 450 nm (OD450) was measured with a 96-well plate reader (EnVision2105, PerkinElmer, USA), to determine the viability of cells.

### Transmission electron microscopy detect the endocytosis of cells

The endocytosis process of cells towards nanoparticles was observed by TEM. 293 T/Hela cells were incubated with 100 μM Si-IONPs for different times (0, 15 min, 30 min, 1 h, 3 h, 6 h, 12 h, 24 h and 48 h), respectively. After incubation, cells were washed with phosphate buffered solution (PBS) for three times and fixed with 3% glutaraldehyde solution subsequently. Samples were dehydrated and embedded into Spurr’s resin, and then, cut into 90 nm ultrathin sections with an ultramicrotome. The TEM images at different processing times were obtained with a TEM (JEM-1230, JEOL, Japan) operating at 80 kV.

### Intracellular nanoparticle content detection using inductively coupled plasma mass spectroscopy

Cells were incubated with 100 μM Si-IONPs for 0/3/6/12/24/48 h, respectively. Then washed cells with PBS thoroughly to remove unabsorbed stray nanoparticles. Cell pellets were then dried under vacuum condition and digested with HNO_3_ (65%) at 150°C. Subsequently, samples were diluted with double distilled water at 1:10 and the nanoparticle content was measured using inductively coupled plasma mass spectroscopy (Elemental X7, Thermo Scientific, USA).

### Sample preparation and analysis by liquid chromatography-tandem mass spectrometry

Cells incubated with 100 μM Si-IONPs for different times were resuspended in lysis buffer (50 mM Hepes, 0.4% NP-40, 0.1 mM Na_3_VO_4_, 10 mM MgCl_2_, 1 mM Tcep, 5 mM b-glycerophosphate, 1 × protease inhibitors), freeze and thaw 5 times in liquid nitrogen and cell lysates were centrifuged at 16 000 *g* for 15 min at 4°C. The protein concentrations in supernatant were determined using bicinchoninic acid (BCA) protein assay kit (CW0014S, CWBIO, China), each sample was quantified to 50 μg, denatured and reduced by adding 100 mM TEAB, 20 mM TCEP and 5% TFE and incubated for 30 min at 55°C, followed by alkylation with CAA (55 mM) added in and incubated in the dark for 30 min at room temperature. Samples were sequentially treated with LysC (50:1, m/m) for 4 h and trypsin (50:1, m/m) overnight at 37°C for digestion, next day samples were desalted by Oasis HLB Extraction Cartridge (Waters, USA). Peptides of each sample were eluted with 1% formic acid in 80% acetonitrile, and spined dry with a centrifugal vacuum evaporator. Finally, peptides were resuspended in buffer containing 1% formic acid (FA) and 1% acetonitrile (ACN), and analyzed by liquid chromatography-tandem mass spectrometry (LC-MS/MS) using an Ultimate 3000 RSLC nano-LC coupled with an Orbitrap Fusion Lumos MS (Thermo Scientific, USA).

### Proteome data analysis

The LC-MS/MS raw data were processed through Proteome Discovery 2.4 software. Data were searched against UniProtKB human FASTA database (Homo sapiens uniport 2022.02, 20371 sequences), the false discovery rate (FDR) on PSMs was set at 1% in protein identification and the obtained proteome datasets were analyzed using R software package (version4.1.1). DEPs were identified with ‘limma’ packed (Version 3.54.2) with *P* values <0.05 and Fold Change (FC) value over 1.5. ‘Mfuzz’ package (Version 2.58.0) was used for cluster analysis of all DEPs in each cell line. The Gene Ontology (GO) and Kyoto Encyclopedia of Genes and Genomes (KEGG) enrichment of DEPs were performed using the ‘clusterprofiler’ package (version 3.18.1).

### Western blotting

Western blotting was performed to indicate the effect of IONPs treatment. Samples were mixed with loading buffer and boiled at 96°C for 10 min before separated via SDS-PAGE gel. Then the proteins were wet transferred onto PVDF membranes (Millipore) and blocked with 5% skim milk solution, followed by the incubation with different primary antibodies (details for antibodies were showed in Table S2) and secondary antibody, respectively. Subsequently the protein bands were visualized using TM High-sig ECL Substrate (Tanon, China).

### Catalase activity detection assay

The catalase assay kit (Nanjing Jiancheng, China) was used to monitor the activity of catalase (CAT) protein. Lysate of both two cells (300 μg) were added into a 96-well plate, respectively, and the CAT detection buffer was then added in sequence. For the negative control, the random sample lysate was added last. Then followed by the detection of absorbance at 405 nm with a multimode plate reader (PerkinElmer, USA). The measurements were carried out in triplicate.

### ROS assay

ROS were detected by flow cytometry using the fluorescence probe DCFH-DA (Sigma-Aldrich). 2 × 10^5^/ml 293 T/Hela cells were cultured in 6-well plates and treated with Si-IONPs at different times, then incubated with 5 μM DCFH-DA in serum-free DMEM in the dark for 30 min; remove the medium and wash cells with serum-free DMEM three times to fully remove the extracellular free DCFH-DA. Harvested the cells, single-cell suspension was prepared and quantified using a FACSort Flow Cytometer (Beckman Coulter, Brea, CA, USA) at 488 nm. The emission of DCF was measured at 515–545 nm. A suitable forward light scatter threshold was used to eliminate cell debris from the investigation.

For analysis of ROS by confocal fluorescence microscopy, cells were seeded in 3 cm dish that had been pre-treated with 1 × polylysine, and treated with Si-IONPs. After different exposure time, cells were incubated with 1 μg/ml Hoechst 33342 and 5 μM DCFH-DA in serum-free DMEM in the dark for 30 min, then, the medium were removed and washed with serum-free DMEM for three times to fully remove the extracellular free Hoechst 33342 and DCFH-DA. All images were captured using a confocal fluorescence microscope (High Speed Confocal Platform, Dragonfly 200, ANDOR, UK).

### Statistical analysis

All experiments were independently repeated three times; data were analyzed using GraphPad Prism 8.0 and presented as mean ± SD. One-way ANOVA test was used to show the statistical difference. *P* < 0.05 was categorized as significance among different groups.

## Results

### The cytotoxicity of Si-IONPs to cancer and normal cells

**Figure 1.**
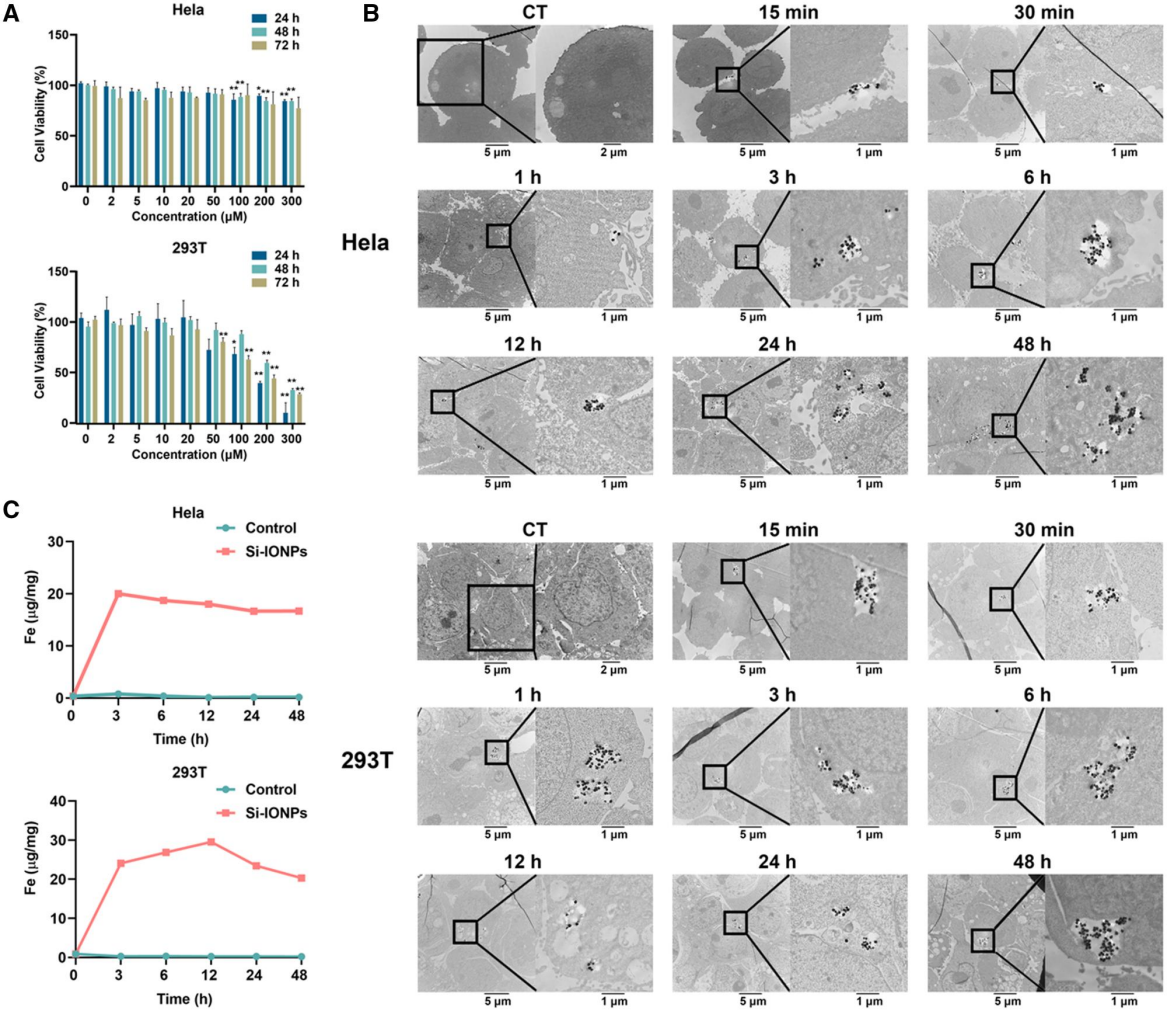
The cytotoxicity, cellular uptake observation and quantification of Si-IONPs on Hela and 293 T cells. (**A**) CCK-8 assay for the cell viability of Hela and 293 T cells at 24/48/72 h treated with 0/2/5/10/20/50100/200/300 μM Si-IONPs. (**B**) TEM images of Hela and 293 T cells after treated with 100 μM Si-IONPs at 0/15/30 min and 1/3/6/12/24/48 h. (**C**) Iron contents in Hela and 293 T cells after treated with 100 μM Si-IONPs at 0/3/6/12/24/48 h. Data were expressed as means ± SD (*n* = 3). *P < 0.05, **P < 0.01, compared to control group.

The cytotoxicity of Si-IONPs to Hela and 293 T cells were detected using CCK8 assay. Cells were treated with the concentration gradient of Si-IONPs for different times, and the results are shown in Figure 1A. The viability of 293 T cells decreased significantly with the increase of Si-IONPs concentration and treatment time, while that of Hela cells showed no obviously change in all concentration at each time point. This indicated that Hela cells have stronger tolerance to Si-IONPs and probably possess a detoxification mechanism which 293 T cells lacked. More elaborate cellular stress responses deserve to be explored in Hela cells.

### Cellular uptake of Si-IONPs

After incubated Hela and 293 T cells with 100 μM Si-IONPs in culture medium at 37°C for different times (0/15/30 min, 1/3/6/12//24//48 h), TEM was used to assess the *in vitro* absorption and location of Si-IONPs. Intracellular Si-IONPs could be observed in Hela cells after incubation for 1 h while that in 293 T cells was only 30 min, indicating that the endocytosis of Si-IONPs in 293 T cells was much faster than that in Hela cells (Figure 1B).

### The dynamic change of intracellular Si-IONPs content

The Si-IONPs content in cells was detected using inductively coupled plasma mass spectrometry which can accurately quantify iron content accumulated in cells, tissues and organs with high sensitivity [21]. As shown in Figure 1C, in Hela cells, the absorption of Si-IONPs reached maximum value of 20.026 μg iron per mg dry weight (dw) at 3 h, then, it tends to decline slowly. There was also a significant surge at 3 h (24.083 μg iron per mg dw) in 293 T cells, but then continuously increase till 12 h (29.554 μg iron per mg dw). The results revealed that the endocytosis rate of 293 T cells was faster and stronger than that of Hela cells, which was also consistent with the results of TEM.

### Proteomic analysis of cells response to Si-IONPs

**Figure 2.**
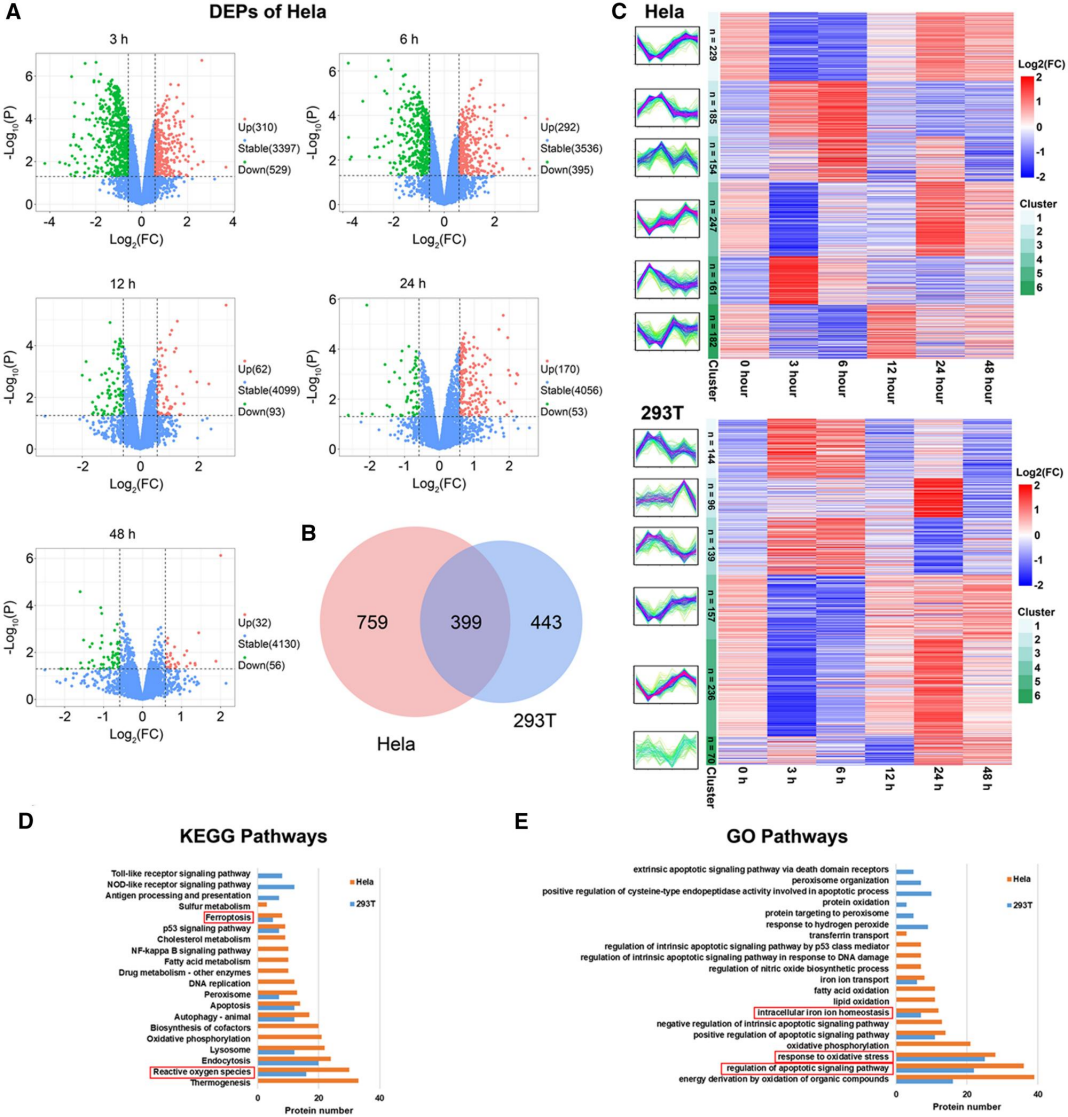
The proteomics analysis of Hela and 293 T cells response to Si-IONPs. (**A**) The volcano plots of DEPs of Hela cells at each timepoint. (**B**) Venn diagram of DEPs in Hela and 293 T cells. (**C**) The heat maps and clusters of all DEPs quantified in Hela and 293 T cells. (**D**) and (**E**) Protein numbers on KEGG and GO pathways enriched by DEPs.

To explore the effects of Si-IONPs on the Hela cancer cells and the 293 T normal cells, liquid chromatography tandem MS (LC-MS/MS) was used to analyze the lysates of these two cells after incubated with 100 μM Si-IONPs for different processing times (0/3/6/12/24/48 h). To investigate the different time-course cellular effects between cancer and normal cells, we compared the protein composition, variation trend and biological functions of all DEPs of Hela and 293 T. Totally 1158 and 842 DEPs were quantified in Hela and 293 T cells, respectively. Volcano plots were carried out to emphasize the variation trend of DEPs of these two cells at different treatment time points (Figure 2A and Figure S1). As shown in Figure 2A, (add total) 839, 687, 155, 223 and 88 DEPs in Hela cells were identified for different processing time, respectively, and 565, 413, 77, 274 and 62 DEPs in 293 T cells were identified (Figure S1), respectively. Venn diagram was performed to visualized the heterogeneity of DEPs in Hela and 293 T cells (Figure 2B). Besides, after Mfuzz clustering analysis of these DEPs, six clusters were obtained for each type of cells, and the heat maps are shown in Figure 2C. In both types of cells DEPs gradually stabilized at 48 h, approaching that of the control group. Surprisingly, there was significantly less DEPs in both Hela and 293 T cells at 12 h in heat maps and clusters distributions which were consistent with the volcano plots.

### DEPs in cells treated with Si-IONPs

**Figure 3.**
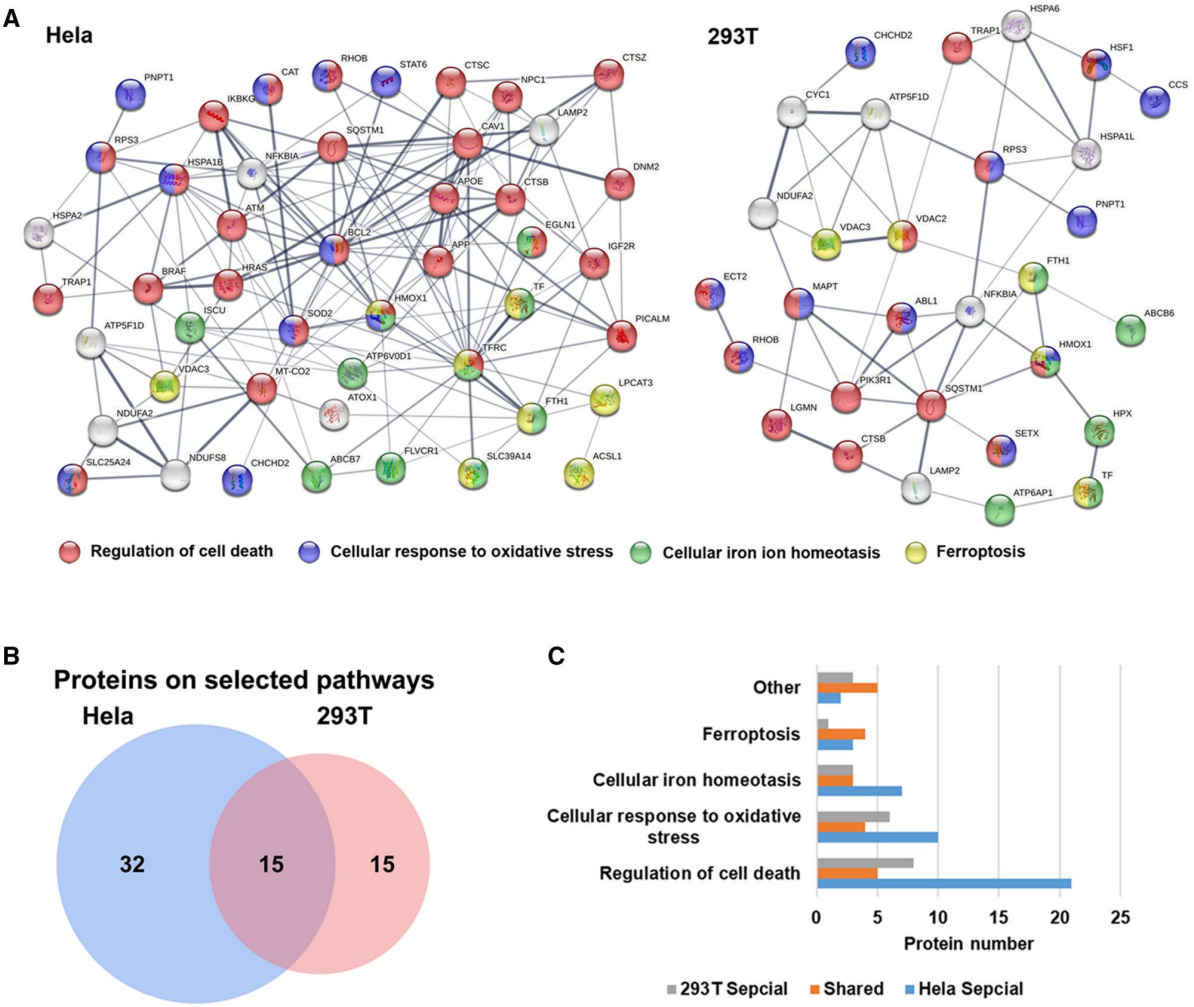
The comparison of DEPs in Hela and 293 T cells involved in oxidative stress, iron ion homeostasis, apoptosis and ferroptosis signaling pathways. (**A**) PPI analysis of DEPs on these pathways. (**B**) Venn map depicting the same DEPs on these pathways and Hela and 293 T cells. (**C**) Protein numbers of DEPs on each pathways n Hela and 293 T cells.

In our study, both KEGG and GO enrichment and analysis of key protein pathways were performed, and the results are displayed in Figure 2D and E, a more complete summary of signaling pathways enriched in KEGG and GO analysis were listed in Tables S3 and S4. KEGG pathway analysis demonstrated that some pathways like drug metabolism enzymes, fatty acid metabolism, DNA replication, DNA mismatch repair, Nucleotide excision repair, oxidative phosphorylation and thermogenesis were unique to Hela cells. Apoptosis, peroxisome, endocytosis, ferroptosis and oxidative stress were both obtained in two kinds of cells while the number of DEPs of these pathways was remarkable higher in Hela cells than in 293 T cells, suggesting that Hela cells may have a better regulatory ability to against the Si-IONPs addition than 293 T cells (Figure 2D and Table S3). In Hela cells, DEPs were mainly involved in biological pathways such as oxidative stress, fatty acid oxidation, iron ion homeostasis, lipid oxidation, oxidative phosphorylation, iron ion transport, regulation of apoptotic signaling pathway in response to DNA damage and transferrin transport based on the GO pathway analysis (Figure 2E). Some pathways such as oxidative stress, iron ion homeostasis and apoptosis were also displayed in 293 T cells, but the number of DEPs in these signaling pathways was much less than that in Hela cells (Figure 2E). Moreover, DEPs in signaling pathways associated with cell population proliferation, positive regulation of cell cycle, cell cycle DNA replication and cellular homeostasis were obtained in Hela cells only depending on GO analysis (Table S4), suggesting that there were more complex and efficient mechanisms of cell damage response and repair in Hela cells. With the analysis and comparison of DEPs in two types of cells, we finally decided to select the DEPs of oxidative stress, iron ion homeostasis, apoptosis and ferroptosis signaling pathways for protein–protein interaction (PPI) network analysis. The STRING database [22] was used to present the results, as shown in Figure 3A. In Hela cells, a much larger and more complicated PPI network was obtained than in 293 T cells. The DEPs numbers of these four signaling pathways in Hela and 293 T cells were 47 and 30, respectively, and there is 15 of DEPs were acquired in both cells (Figure 3B), the proteins in each pathway are listed in Table S4. The heat maps and clusters of the total 62 DEPs involved in these four pathways in two cells are displayed in Supplementary Figure S2. In both types of cells, most of the DEPs were concentrated in the cell death regulation pathway, and that were more abundant in Hela cells (Figure 3C). The DEPs of both cells in ferroptosis, oxidative stress and iron homeostasis pathways accounted for a large proportion (Figure 3C). Therefore, we then selected proteins from these common signaling pathways for further mechanistic verification.

### Si-IONPs induce the proteins expression fluctuation in cellular pathways related to oxidative stress and ferroptosis pathways

Oxidative stress and ferroptosis are both the important pathways that can induce cell death, and there is an obvious causal relationship between each other [23]. It was reported that IONPs added in cells can induce excess ROS which cause oxidative stress and lipid peroxidation, ultimately leads to ferroptosis [12]. In this study, we found that the oxidative stress, iron homeostasis and ferroptosis related proteins such as ferritins, heme oxygenase (HMOX1), tumor necrosis factor receptor-associated protein 1 (TRAP1) and catalase (CAT) were affected visibly after the addition of Si-IONPs both in tumor and normal cells (Figure 3). Ferritins are the only proteins that can store irons in the cell and consist of two subunits, the heavy (Ferritin Heavy Chain (FTH)) and the light (Ferritin light Chain (FTL)) chain [24]. In this study, ferritin is mainly presented in the form of FTH. We confirmed that all the proteins above were associated with oxidative stress, iron homeostasis and ferroptosis signaling pathways (Figure 3A), so we next examined the expression of these proteins.

**Figure 4.**
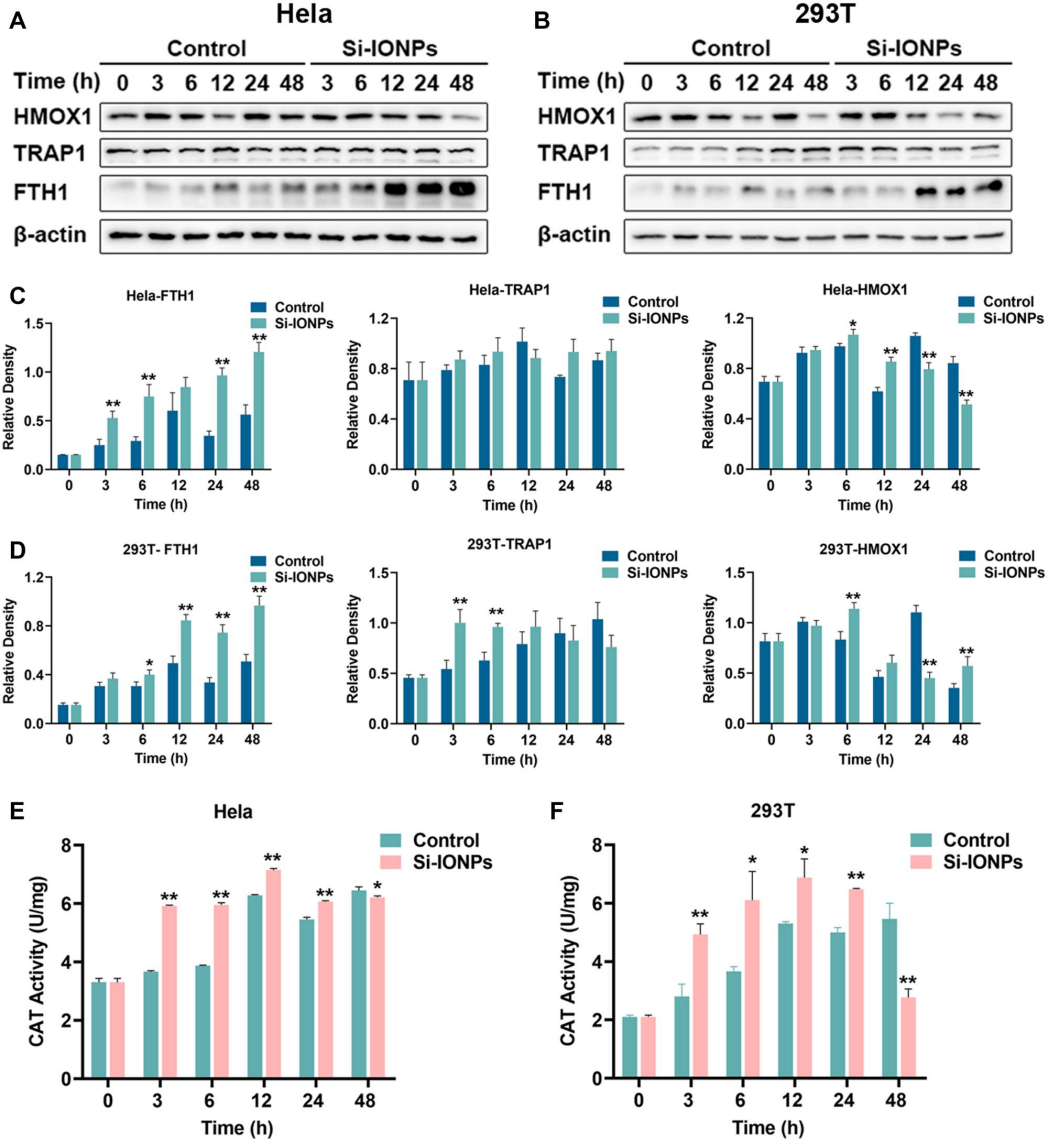
The effects of Si-IONPs on Hela and 293 T cells related to oxidative stress and ferroptosis pathways. (**A**) and (**C**) The expression and histogram changes of HMOX1, TRAP1 and FTH1 in Hela cells were determined by Western blotting with or without 100 μM Si-IONPs treatment at 3/6/12/24/48 h. (**B**) and (**D**) The expression and histogram changes of HMOX1, TRAP1 and FTH1 in 293 T cells were determined by Western blotting with or without 100 μM Si-IONPs treatment at 3/6/12/24/48 h. (**E**) and (**F**) CAT activity after 100 μM Si-IONPs treatment at each timepoint. Data were expressed as means ± SD (*n* = 3). *P < 0.05, **P < 0.01, compared to control group.

We measured the expression levels of FTH, HMOX1 and TRAP1 in Hela and 293 T cells which were incubated with Si-IONPs for different times. Interestingly, the data indicated that Si-IONPs incubation led to the significant fluctuations in protein expression over the time-course in Hela cells, especially in the FTH1, which increased obviously after the Si-IONPs added in (Figure 4A and C). This may because the addition of Si-IONPs led to the surge of iron ions in the cell, the production of ROS and the excessive upregulation of FTH1, which is a cytoprotective mechanism to bind to free iron and scavenge the free radicals. Different from the above, with the extension of treatment time, FTH1 in control group had a slight increase trend, and the highest reached at 12 h, which may be related to the iron metabolic requirement of cells, and the ferritin was down-regulated after reaching the sufficient amount of iron at 12 h (Figure 4A and C). The variation tendency of HMOX1 was completely disparate, which tended to increase at first and then decreased at 12 h with the incubation of Si-IONPs. The initial augment of HMOX1 helps cells cope with oxidative stress caused by Si-IONPs, and subsequently, increasing ferrous iron content inhibited its expression. In control group, HMOX1 showed unexplained fluctuation which plummeted at 12 h, increased again at 24 h and as well decreased at 48 h (Figure 4A and C). This may correspond to the changes in FTH1. The variation trend of FTH1 and HMOX1 in 293 T were similar to Hela, and the variation trend of HMOX1 was more dramatic than in Hela cells, suggesting that Si-IONPs had a greater effect on 293 T cells (Figure 4B and D). Coincidently, there were no significant changes on TRAP1 in Hela cells while an obvious differences were acquired in 293 T. However, the trend of TRAP1 in both two cell types were inconsistent with FTH1 (Figure 4). We also measured the activity of CAT in Hela and 293 T cells, and showed the variation trend of falling after rising, which agreed with the variation of HMOX1 and revealed the cell response to ROS. Also, the change tendency of CAT activity was more significant in 293 T cells (Figure 4E and F). The above results showed that both kinds of cells exhibited antioxidant stress to repair ROS damage after the Si-IONPs stimulation, but 293 T cells were more sensitive to Si-IONPs than Hela cells.

### Si-IONPs induce the ROS in both two cells

**Figure 5.**
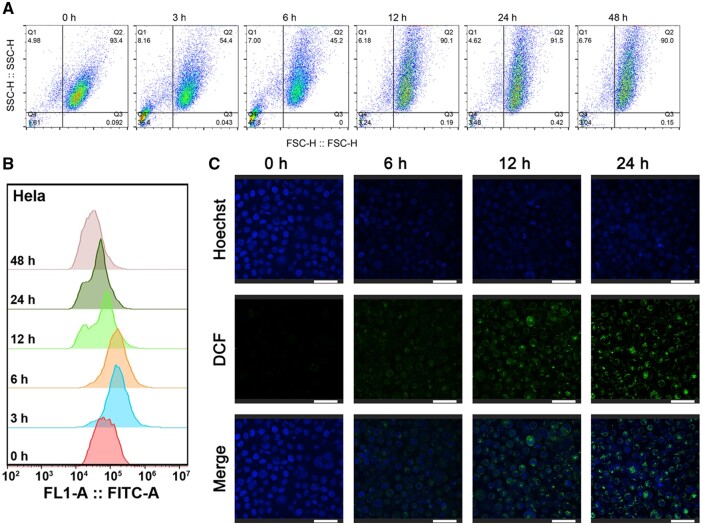
Si-IONPs induced ROS in Hela cells. (**A**) and (**B**) Flow cytometry revealed the cells’ status and ROS levels changes after 100 μM Si-IONPs treatment. (**C**) Confocal fluorescence microscopy visualized the ROS levels changes after 100 μM Si-IONPs treatment (Scale bar = 50 μm).

**Figure 6.**
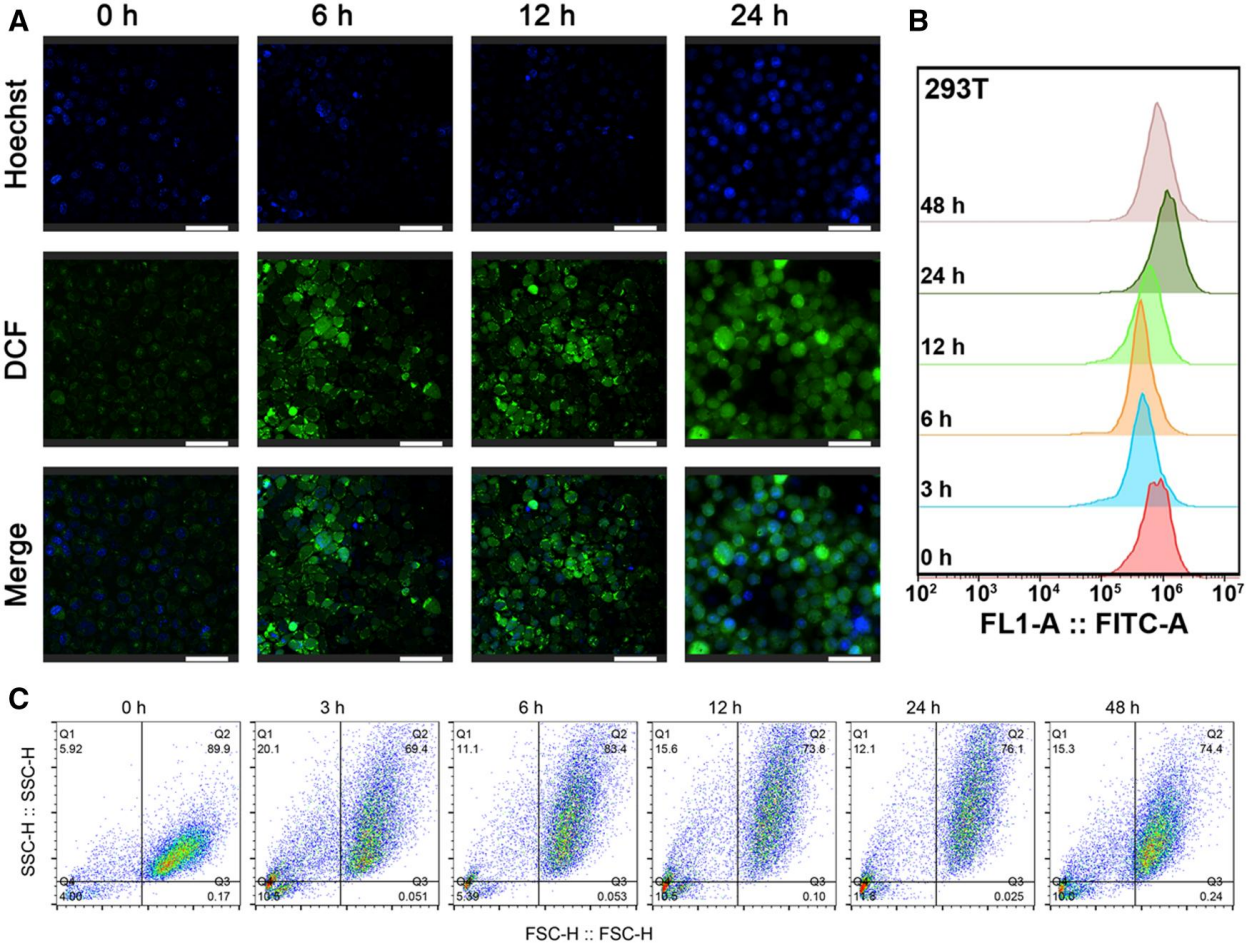
Si-IONPs induced ROS in 293 T cells. (**A**) Confocal fluorescence microscopy visualized the ROS levels changes after 100 μM Si-IONPs treatment. (**B**) and (**C**) Flow cytometry revealed the ROS levels changes and cells’ status after 100 μM Si-IONPs treatment (Scale bar = 50 μm).

To more intuitively monitor the ROS levels, we performed flow cytometry and confocal fluorescence imaging to verify the ROS levels induced by Si-IONPs in both Hela and 293 T cells. As shown in Figure 5, both flow cytometry and confocal fluorescence images indicated that Si-IONPs induced a slight ROS variation in Hela cells, which reflected that Si-IONPs didn't have serious effect on the Hela cells or a strong self-regulation ability existed in Hela cells. However, Si-IONPs led to significant ROS changes in 293 T cells, and even we could not focus the images after 24-h treatment with Si-IONPs (Figure 6). The corresponding flow cytometry and confocal fluorescence imaging results of ROS were consistent with the conclusion above and showed that Hela cells recovered more easily after Si-IONPs interference than the normal cells 293 T.

These results above indicated that the addition of Si-IONPs produced excess iron ions and led to ROS generation, inducing cells to regulate the expression of oxidative stress, iron homeostasis and ferroptosis related proteins such as FTH1, HMOX1, TRAP1 and CAT. In addition, compared with Hela cells which exhibited strong self-regulation ability, Si-IONPs had a more significant effect on 293 T cells and were more likely to cause cytotoxicity in normal cells.

## Discussion

Since the 1990s, the incidence of cancer has increased dramatically worldwide. IONPs are emerging materials that have shown fast development and excellent application value. They have been widely employed in many fields of biomedicine, especially in cancer therapy. The main application areas of IONPs in cancer treatment are magnetic hyperthermia, contrast agents for MRI, chemotherapeutic agent delivery, and even themselves exhibit cytotoxicity on cancer cells. Recently, more and more researches revealed that IONPs could induce tumor cell death through causing oxidative stress, DNA strand breakage, damaging the integrity of intracellular membrane, inducing ferroptosis and so forth [4–6]. It provides a new direction for antitumor therapy which needs more data on biosafety evaluation and more study on the underlying mechanisms of IONPs to maximize their therapeutic effectiveness and safety.

IONPs has been reported to induce cancer cell death via triggering oxidative stress, breaking iron homeostasis and resulting in ferroptosis. The ferrous or ferric ions that released from IONPs degradation in cells can lead to Fenton reaction, which in turn contributing to the oxidative stress, ROS generation, lipid peroxidation and ferroptosis [12]. Although many kinds of different coated IONPs have been used in antitumor studies and tested to induce cancer cell death via triggering oxidative stress and ferroptosis, there are still insufficient data on the safety evaluation and the underlying mechanism of IONPs to induce cell death, especially in the part of continuous time monitoring of tumor cell response to IONPs. Here, we monitored the time-course, identified and quantified the DEPs of Hela cells and 293 T cells treated with Si-IONPs using mass spectrometry-based proteomics. We compared the differences of protein composition, allocation and physical characteristics between these two kinds of cells and measured the expression of proteins associated with oxidative stress and ferroptosis pathways in these two kinds of cells. The major findings of this study pointed out: ①Cell death occurred in both types of cells after Si-IONPs treatment, and the death of 293 T cells were more significant than that of Hela cells; ②Electron microscopy showed that Si-IONPs endocytosis rate of Hela cells was slower than 293 T. ③The results of MS-based proteomics showed significant changes in the signaling pathways related to oxidative stress, iron homeostasis, apoptosis, immune response, cell cycle and ferroptosis. ④Further in-depth studies on oxidative stress, iron homeostasis and ferroptosis pathways showed that the expression levels of related proteins such as FTH1, HMOX1 and TRAP1 significantly changed with the treatment of Si-IONPs in time-course, and the CAT enzyme activity also markedly changed, especially in 293 T cells. The results of flow cytometry also showed the notably production of ROS after Si-IONPs incubation in both cells and Hela cells recovered more easily after Si-IONPs interference than 293 T. These findings revealed that Si-IONPs could induce cell death by inducing apoptosis, disrupting intracellular iron homeostasis, causing oxidative stress, ROS and even resulting in ferroptosis. In addition, Hela cells were more tolerant to Si-IONPs than 293 T cells, which may be partly due to their different endocytosis modes. 293 T endocytosis of Si-IONPs was faster, IONPs triggered more prominent cytotoxicity in 293 T cells.

In this study, we compared the cytotoxicity of Si-IONPs to cancer cells (Hela) and normal cells (293 T), to reveal the in-depth mechanisms of Si-IONPs antitumor property and provide safety data for the application of Si-IONPs. Different cell types show various endocytosis rate, intracellular dynamic tracking trajectory and uptake capacity to nanoparticles [25]. Consistently, our TEM results suggested a significant difference in endocytosis rates of the two cells. 293 T cells had a faster and higher endocytosis rate for Si-IONPs, which may cause it to die more easily than Hela cells (Figure 1). To investigate the dynamic biological processes and the DEPs of cells affected by Si-IONPs, MS-based proteomics was used here and we found that two cell types showed both similarities and differences in response to Si-IONPs. Compared with 293 T cells, Hela cells had more differential signaling pathways and its DEPs were mainly involved in oxidative stress, fatty acid oxidation, iron ion homeostasis, oxidative phosphorylation, immune response, DNA double-strand break repair, lipid metabolism and apoptosis based on the GO pathway analysis. Some pathways such as oxidative stress, ferroptosis, immune response and apoptosis were also displayed based on KEGG and were detected in 293 T cells (Figure 2), combined with the existing research status, we then focused on the analysis and detection of differentially expressed proteins in these pathways.

Ferritins are the only proteins that can store irons in the cell and play an important role in iron metabolism. Ferritin consists of two subunits, the heavy (FTH) and the light (FTL) chain [24]. In this study, ferritin is mainly presented as the form of FTH identified throughout all the timepoints. Nowadays, FTH was considered to be involved in the apoptosis and ferroptosis [26]. Surprisingly, the data indicated that Si-IONPs incubation led to the more significant augment in protein expression of FTH1 over the time-course in Hela cells than 293 T (Figure 4). Decreased expression of FTH1 led to the accumulation of iron in cell, which in turn resulted in Fenton reaction, ROS generation and ferroptosis [27]. So, the sharply increased expression of FTH1 in Hela cells reflected its stronger stress ability than 293 T cells, and Si-IONPs exhibit more toxic to 293 T, which was consistent with the cytotoxicity test and the electron microscope results above.

HMOX1 is an important regulator which can degrade heme and release ferrous iron, maintain intracellular iron homeostasis. HMOX1 has been reported to be participated in oxidative stress and its upregulation increases the ROS and facilitates ferroptosis when there is no additional iron intervention in various diseases [28–33]. Interestingly, in our study, a remarkable fluctuation was detected and HMOX1 tended to increase at first and then decreased at the 12th h with the incubation of Si-IONPs in both types of cells, especially in 293 T cells, and finally it increased again in 293 T cells (Figure 4). The initial augment of HMOX1 helped cells cope with oxidative stress caused by Si-IONPs, and subsequently, increasing ferrous iron from Si-IONPs inhibited its expression. The fluctuation of HMOX1 protein expression reflected the change of iron content in cells caused by Si-IONPs.

TRAP1 is mitochondrial heat shock protein 75-kDa (Hsp75), belongs to the Hsp90 family and has been reported with the ability to offset oxidative stress in cancer cells, its overexpression reduces ROS production and protects cells from oxidative stress-mediated damage [24, 34–37]. Interestingly, in our study, there was a remarkable change of TRAP1 expression in 293 T cells that decreased with Si-IONPs incubation while increased in the control group. The data indicated that Si-IONPs could weaken the ability of 293 T cells to response ROS. Curiously, slight difference was detected in Hela cells in which TRAP1maintained a relatively high level of expression. We also measured the activity of CAT in Hela and 293 T cells. CAT is an enzymatic antioxidant which can remove H_2_O_2_ and reduce ROS damage to cells [38]. In our study, the data showed the variation trend of falling after rising in 293 T cells. The activity of CAT in Hela cells was significantly higher than that in 293 T cells, which agreed with the variation of HMOX1 and revealed the cell response to ROS. The corresponding flow cytometry results of ROS also showed this conclusion.

These results above indicated that the addition of Si-IONPs produce excess ROS production, inducing stress response and remarkable protein expression changes in both two types of cells, while Hela had a stronger self-repair ability than 293 T.

**Figure 7.**
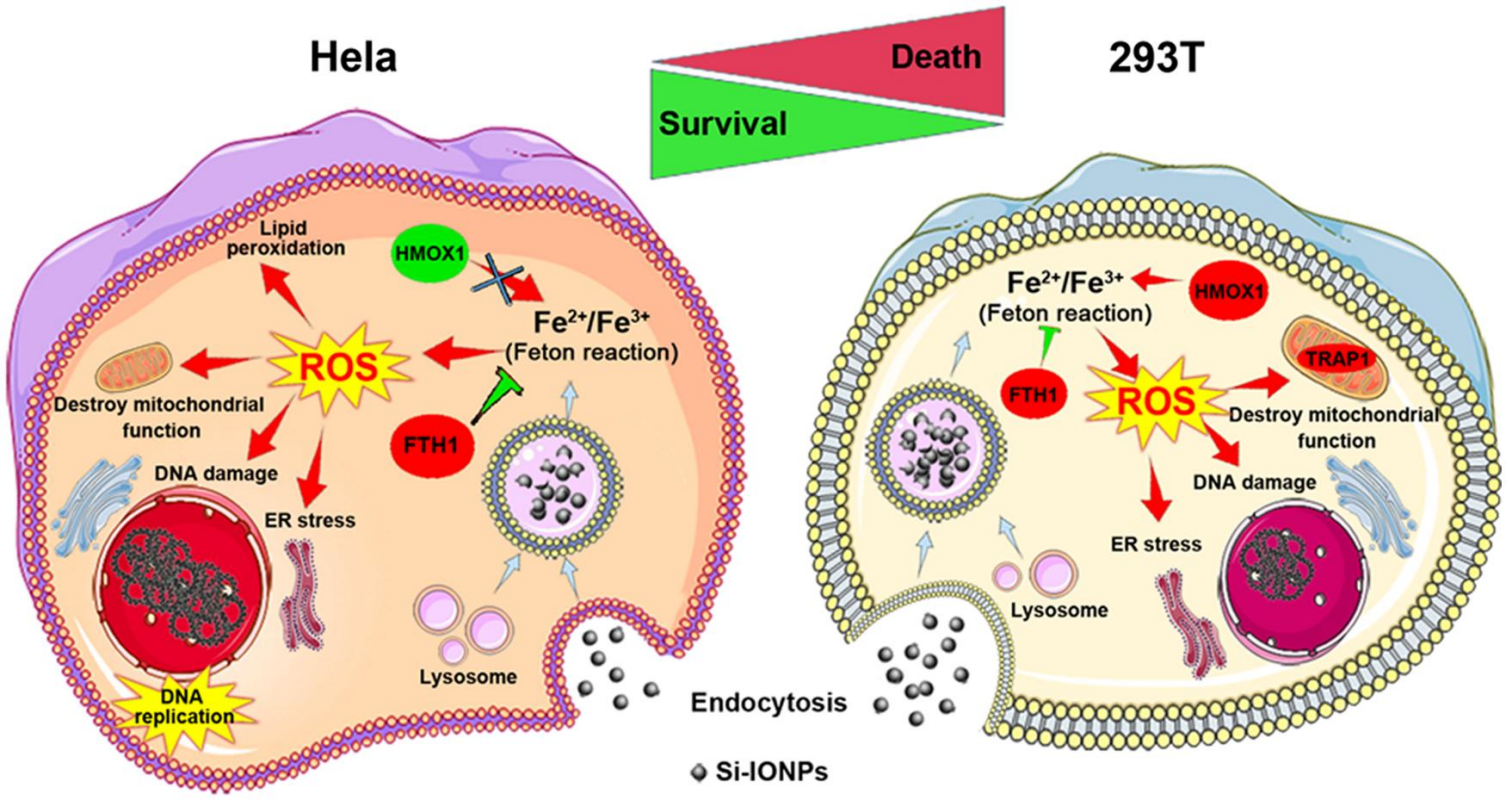
Schematic diagram showing the mechanisms of apoptosis regulation by silica-coated iron oxide nanoparticles (Si-IONPs) in Hela and 293 T cells. Si-IONPs significantly increases the content of iron ions in cells, causing Fenton reaction and inducing ROS, which can further cause endoplasmic reticulum stress, DNA breakage, interference with mitochondrial function, lipid peroxidation and ultimately cell death. In Hela cells, the cells themselves regulate the significant overexpression of FTH1 and decrease the expression of HMOX1 in response to the increase of iron ions and Fenton reaction, and regulate the cell cycle to promote cell proliferation. The lack of self-regulatory mechanisms in 293 T cells, as in Hela, ultimately leads to cell death.

In addition to the above DEPs, in Hela cells, we also found significant changes in many members of the ATP-binding cassette (ABC) transporters which make cancer cells resistant to chemotherapy agents [39], such as ABCB10, ABCB7, ABCD1, ABCE1, ABCF1, ABCF2, ABCF3 and ABCG2, and only ABCF1, ABCF2 and ABCF3 were detected in 293 T cells. Moreover, DEPs in signaling pathways associated with cell population proliferation, positive regulation of cell cycle, cell cycle DNA replication and cellular homeostasis were obtained in Hela cells only depending on GO analysis (Table S4), suggesting that there were more complex and efficient mechanisms of cell damage response and repair in Hela cells. Furthermore, the number of DEPs related to the DNA damage response, apoptosis regulation and transferrin transport pathways in Hela cells was obviously higher than that in 293 T cells, these data all indicated that Hela had stronger resistance than 293 T (Figure 7).

With the increasing application of IONPs in the biomedical field, their biosafety has been widely concerned. They were the only biodegradable metal nanoparticles approved for clinical use by the US FDA, but nowadays a number of initially approved IONP-based nanomedicines have been withdrawn due to their adverse effects or poor clinical performance [40]. IONPs were reported passively accumulated in different liver, spleen and lymph nodes, causing endoplasmic reticulum stress and lysosomal dysfunction, and eventually leading to cell apoptosis [13, 14, 41, 42]. Studies have shown that some kind of IONPs retained ∼11% of injected dose in liver up to 84 days post intravenous injection, and this long-lasting phenomenon could not be affected by the sizes of IONPs [43–46].

Unlike the dietary iron, in which generally does not cause toxic effects to the body, pharmaceutical iron (such as ferumoxsil and ferumoxide) will lead to iron overload in organisms in a short time, causing gastrointestinal discomfort and damage to liver, heart, brain and lungs [47]. Moreover, the toxic effects of IONPs have been discovered in animals with various exposure routes [48–50]. Although IONPs in tissues could be degraded into ferrous or ferric ions, excessive accumulation of intracellular irons resulted in the generation of ROS, DNA breakage and even other serious damages. Thus, more studies on the specific mechanisms especially involved in or interfered with physiological iron metabolism after degradation need to be carried out in depth.

## Conclusion

In conclusion, compared with cancer cells, nanoparticles are more likely to induce normal cell death, and cancer cells have their own unique mechanisms to defense invaders, reminding scientists that future *in vivo* and *in vitro* studies of nanoparticles need to be cautious, and more safety data are needed for further clinical treatment.

## Supplementary Material

rbae065_Supplementary_Data

## Data Availability

The authors confirm that all data are available within the article and its supplementary materials. The raw data are available from the corresponding author upon reasonable request.

## References

[1] Feng Q , Liu Y, Huang J, Chen K, Huang J, Xiao K. Uptake, distribution, clearance, and toxicity of iron oxide nanoparticles with different sizes and coatings. Sci Rep 2018;8:2082. 29391477 10.1038/s41598-018-19628-z PMC5794763

[2] Dadfar SM , Roemhild K, Drude NI, von Stillfried S, Knüchel R, Kiessling F, Lammers T. Iron oxide nanoparticles: diagnostic, therapeutic and theranostic applications. Adv Drug Deliv Rev 2019;138:302–25. 30639256 10.1016/j.addr.2019.01.005 PMC7115878

[3] Mao Z , Li X, Wang P, Yan H. Iron oxide nanoparticles for biomedical applications: an updated patent review (2015-2021). Expert Opin Ther Pat 2022;32:939–52. 35929879 10.1080/13543776.2022.2109413

[4] Chen H , Wen J. Iron oxide nanoparticles loaded with paclitaxel inhibits glioblastoma by enhancing autophagy-dependent ferroptosis pathway. Eur J Pharmacol 2022;921:174860. 35278406 10.1016/j.ejphar.2022.174860

[5] Alarifi S , Ali D, Alkahtani S, Alhader MS. Iron oxide nanoparticles induce oxidative stress, DNA damage, and caspase activation in the human breast cancer cell line. Biol Trace Elem Res 2014;159:416–24. 24748114 10.1007/s12011-014-9972-0

[6] Iliasov AR , Nizamov TR, Naumenko VA, Garanina AS, Vodopyanov SS, Nikitin AA, Pershina AG, Chernysheva AA, Kan Y, Mogilnikov PS, Metelkina ON, Schetinin IV, Savchenko AG, Majouga AG, Abakumov MA. Non-magnetic shell coating of magnetic nanoparticles as key factor of toxicity for cancer cells in a low frequency alternating magnetic field. Colloids Surf B Biointerfaces 2021;206:111931. 34171621 10.1016/j.colsurfb.2021.111931

[7] Guan Q , Guo R, Huang S, Zhang F, Liu J, Wang Z, Yang X, Shuai X, Cao Z. Mesoporous polydopamine carrying sorafenib and SPIO nanoparticles for MRI-guided ferroptosis cancer therapy. J Control Release 2020;320:392–403. 32004587 10.1016/j.jconrel.2020.01.048

[8] Zheng DW , Lei Q, Zhu JY, Fan JX, Li CX, Li C, Xu Z, Cheng SX, Zhang XZ. Switching apoptosis to ferroptosis: metal-organic network for high-efficiency anticancer therapy. Nano Lett 2017;17:284–91. 28027643 10.1021/acs.nanolett.6b04060

[9] Kim SE , Zhang L, Ma K, Riegman M, Chen F, Ingold I, Conrad M, Turker MZ, Gao M, Jiang X, Monette S, Pauliah M, Gonen M, Zanzonico P, Quinn T, Wiesner U, Bradbury MS, Overholtzer M. Ultrasmall nanoparticles induce ferroptosis in nutrient-deprived cancer cells and suppress tumour growth. Nat Nanotechnol 2016;11:977–85. 27668796 10.1038/nnano.2016.164 PMC5108575

[10] Ou W , Mulik RS, Anwar A, McDonald JG, He X, Corbin IR. Low-density lipoprotein docosahexaenoic acid nanoparticles induce ferroptotic cell death in hepatocellular carcinoma. Free Radic Biol Med 2017;112:597–607. 28893626 10.1016/j.freeradbiomed.2017.09.002 PMC5848495

[11] Fernández-Acosta R , Iriarte-Mesa C, Alvarez-Alminaque D, Hassannia B, Wiernicki B, Díaz-García AM, Vandenabeele P, Vanden Berghe T, Pardo Andreu GL. Novel iron oxide nanoparticles induce ferroptosis in a panel of cancer cell lines. Molecules 2022;27:3970. 35807217 10.3390/molecules27133970 PMC9268471

[12] Zhang Y , Gao X, Yan B, Wen N, Lee WSV, Liang XJ, Liu X. Enhancement of CD8^+^ T-cell-mediated tumor immunotherapy via magnetic hyperthermia. ChemMedChem 2022;17:e202100656. 34806311 10.1002/cmdc.202100656

[13] Hamm B , Staks T, Taupitz M, Maibauer R, Speidel A, Huppertz A, Frenzel T, Lawaczeck R, Wolf KJ, Lange L. Contrast-enhanced MR imaging of liver and spleen: first experience in humans with a new superparamagnetic iron oxide. J Magn Reson Imaging 1994;4:659–68. 7981510 10.1002/jmri.1880040508

[14] Baiu DC , Brazel CS, Bao Y, Otto M. Interactions of iron oxide nanoparticles with the immune system: challenges and opportunities for their use in nano-oncology. Curr Pharm Des 2013;19:6606–21. 23621531 10.2174/13816128113199990409

[15] Jiang Z , Shan K, Song J, Liu J, Rajendran S, Pugazhendhi A, Jacob JA, Chen B. Toxic effects of magnetic nanoparticles on normal cells and organs. Life Sci 2019;220:156–61. 30716338 10.1016/j.lfs.2019.01.056

[16] Mendes ML , Dittmar G. Targeted proteomics on its way to discovery. Proteomics 2022;22:e2100330. 35816345 10.1002/pmic.202100330

[17] Rozanova S , Barkovits K, Nikolov M, Schmidt C, Urlaub H, Marcus K. Quantitative mass spectrometry-based proteomics: an overview. Methods Mol Biol 2021;2228:85–116. 33950486 10.1007/978-1-0716-1024-4_8

[18] Lai ZW , Yan Y, Caruso F, Nice EC. Emerging techniques in proteomics for probing nano-bio interactions. ACS Nano 2012;6:10438–48. 23214939 10.1021/nn3052499

[19] Zhang H , Peng J, Li X, Liu S, Hu Z, Xu G, Wu R. A nano-bio interfacial protein corona on silica nanoparticle. Colloids Surf B Biointerfaces 2018;167:220–8. 29656205 10.1016/j.colsurfb.2018.04.021

[20] Meng Y , Chen J, Liu Y, Zhu Y, Wong YK, Lyu H, Shi Q, Xia F, Gu L, Zhang X, Gao P, Tang H, Guo Q, Qiu C, Xu C, He X, Zhang J, Wang J. A highly efficient protein corona-based proteomic analysis strategy for the discovery of pharmacodynamic biomarkers. J Pharm Anal 2022;12:879–88. 36605576 10.1016/j.jpha.2022.07.002 PMC9805947

[21] Mittal M , Kumar K, Anghore D, Rawal RK. ICP-MS: analytical method for identification and detection of elemental impurities. Curr Drug Discov Technol 2017;14:106–20. 28003007 10.2174/1570163813666161221141402

[22] Szklarczyk D , Gable AL, Nastou KC, Lyon D, Kirsch R, Pyysalo S, Doncheva NT, Legeay M, Fang T, Bork P, Jensen LJ, von Mering C. The STRING database in 2021: customizable protein-protein networks, and functional characterization of user-uploaded gene/measurement sets. Nucleic Acids Res 2021;49:D605–12. 33237311 10.1093/nar/gkaa1074 PMC7779004

[23] Zhu J , Xiong Y, Zhang Y, Wen J, Cai N, Cheng K, Liang H, Zhang W. The molecular mechanisms of regulating oxidative stress-induced ferroptosis and therapeutic strategy in tumors. Oxid Med Cell Longev 2020;2020:8810785. 33425217 10.1155/2020/8810785 PMC7772020

[24] Liu J , Ren Z, Yang L, Zhu L, Li Y, Bie C, Liu H, Ji Y, Chen D, Zhu M, Kuang W. The NSUN5-FTH1/FTL pathway mediates ferroptosis in bone marrow-derived mesenchymal stem cells. Cell Death Discov 2022;8:99. 35249107 10.1038/s41420-022-00902-z PMC8898311

[25] Lojk J , Bregar VB, Rajh M, Miš K, Kreft ME, Pirkmajer S, Veranič P, Pavlin M. Cell type-specific response to high intracellular loading of polyacrylic acid-coated magnetic nanoparticles. Int J Nanomed 2015;10:1449–62. 10.2147/IJN.S76134 PMC4340463 25733835

[26] Asperti M , Bellini S, Grillo E, Gryzik M, Cantamessa L, Ronca R, Maccarinelli F, Salvi A, De Petro G, Arosio P, Mitola S, Poli M. H-ferritin suppression and pronounced mitochondrial respiration make hepatocellular carcinoma cells sensitive to RSL3-induced ferroptosis. Free Radic Biol Med 2021;169:294–303. 33892112 10.1016/j.freeradbiomed.2021.04.024

[27] Hu W , Zhou C, Jing Q, Li Y, Yang J, Yang C, Wang L, Hu J, Li H, Wang H, Yuan C, Zhou Y, Ren X, Tong X, Du J, Wang Y. FTH promotes the proliferation and renders the HCC cells specifically resist to ferroptosis by maintaining iron homeostasis. Cancer Cell Int 2021;21:709. 34965856 10.1186/s12935-021-02420-x PMC8717654

[28] Wu D , Hu Q, Wang Y, Jin M, Tao Z, Wan J. Identification of HMOX1 as a critical ferroptosis-related gene in atherosclerosis. Front Cardiovasc Med 2022;9:833642. 35498043 10.3389/fcvm.2022.833642 PMC9046663

[29] Fang X , Wang H, Han D, Xie E, Yang X, Wei J, Gu S, Gao F, Zhu N, Yin X, Cheng Q, Zhang P, Dai W, Chen J, Yang F, Yang HT, Linkermann A, Gu W, Min J, Wang F. Ferroptosis as a target for protection against cardiomyopathy. Proc Natl Acad Sci USA 2019;116:2672–80. 30692261 10.1073/pnas.1821022116 PMC6377499

[30] Hassannia B , Vandenabeele P, Vanden Berghe T. Targeting ferroptosis to iron out cancer. Cancer Cell 2019;35:830–49. 31105042 10.1016/j.ccell.2019.04.002

[31] Lin H , Chen X, Zhang C, Yang T, Deng Z, Song Y, Huang L, Li F, Li Q, Lin S, Jin D. EF24 induces ferroptosis in osteosarcoma cells through HMOX1. Biomed Pharmacother 2021;136:111202. 33453607 10.1016/j.biopha.2020.111202

[32] Meng Z , Liang H, Zhao J, Gao J, Liu C, Ma X, Liu J, Liang B, Jiao X, Cao J, Wang Y. HMOX1 upregulation promotes ferroptosis in diabetic atherosclerosis. Life Sci 2021;284:119935. 34508760 10.1016/j.lfs.2021.119935

[33] Lai X , Sun Y, Zhang X, Wang D, Wang J, Wang H, Zhao Y, Liu X, Xu X, Song H, Ping W, Sun Y, Hu Z. Honokiol induces ferroptosis by upregulating HMOX1 in acute myeloid leukemia cells. Front Pharmacol 2022;13:897791. 35645831 10.3389/fphar.2022.897791 PMC9132251

[34] Zhang X , Dong Y, Gao M, Hao M, Ren H, Guo L, Guo H. Knockdown of TRAP1 promotes cisplatin-induced apoptosis by promoting the ROS-dependent mitochondrial dysfunction in lung cancer cells. Mol Cell Biochem 2021;476:1075–82. 33196942 10.1007/s11010-020-03973-7

[35] Rasola A , Neckers L, Picard D. Mitochondrial oxidative phosphorylation TRAP(1)ped in tumor cells. Trends Cell Biol 2014;24:455–63. 24731398 10.1016/j.tcb.2014.03.005 PMC7670877

[36] Xie S , Wang X, Gan S, Tang X, Kang X, Zhu S. The mitochondrial chaperone TRAP1 as a candidate target of oncotherapy. Front Oncol 2020;10:585047. 33575209 10.3389/fonc.2020.585047 PMC7870996

[37] Avolio R , Matassa DS, Criscuolo D, Landriscina M, Esposito F. Modulation of mitochondrial metabolic reprogramming and oxidative stress to overcome chemoresistance in cancer. Biomolecules 2020;10:135. 31947673 10.3390/biom10010135 PMC7023176

[38] Ali SS , Ahsan H, Zia MK, Siddiqui T, Khan FH. Understanding oxidants and antioxidants: classical team with new players. J Food Biochem 2020;44:e13145. 31960481 10.1111/jfbc.13145

[39] Robey RW , Pluchino KM, Hall MD, Fojo AT, Bates SE, Gottesman MM. Revisiting the role of efflux pumps in multidrug-resistant cancer. Nat Rev Cancer 2018;18:452–64. 29643473 10.1038/s41568-018-0005-8 PMC6622180

[40] Frtús A , Smolková B, Uzhytchak M, Lunova M, Jirsa M, Kubinová Š, Dejneka A, Lunov O. Analyzing the mechanisms of iron oxide nanoparticles interactions with cells: a road from failure to success in clinical applications. J Control Release 2020;328:59–77. 32860925 10.1016/j.jconrel.2020.08.036

[41] Uzhytchak M , Lunova M, Smolková B, Jirsa M, Dejneka A, Lunov O. Iron oxide nanoparticles trigger endoplasmic reticulum damage in steatotic hepatic cells. Nanoscale Adv 2023;5:4250–68. 37560414 10.1039/d3na00071k PMC10408607

[42] Uzhytchak M , Smolková B, Lunova M, Frtús A, Jirsa M, Dejneka A, Lunov O. Lysosomal nanotoxicity: impact of nanomedicines on lysosomal function. Adv Drug Deliv Rev 2023;197:114828. 37075952 10.1016/j.addr.2023.114828

[43] Tate JA , Petryk AA, Giustini AJ, Hoopes PJ. In vivo biodistribution of iron oxide nanoparticles: an overview. Proc SPIE Int Soc Opt Eng 2011;7901:790117. 24478825 10.1117/12.876414 PMC3903270

[44] Tietze R , Jurgons R, Lyer S, Schreiber E, Wiekhorst F, Eberbeck D, Richter H, Steinhoff U, Trahms L, Alexiou C. Quantification of drug-loaded magnetic nanoparticles in rabbit liver and tumor after in vivo administration. J Magn Magn Mater 2009;321:1465–8.

[45] Shanehsazzadeh S , Oghabian MA, Daha FJ, Amanlou M, Allen BJ. Biodistribution of ultra small superparamagnetic iron oxide nanoparticles in BALB mice. J Radioanal Nucl Chem 2013;295:1517–23.

[46] Bourrinet P , Bengele HH, Bonnemain B, Dencausse A, Idee JM, Jacobs PM, Lewis JM. Preclinical safety and pharmacokinetic profile of ferumoxtran-10, an ultrasmall superparamagnetic iron oxide magnetic resonance contrast agent. Invest Radiol 2006;41:313–24. 16481915 10.1097/01.rli.0000197669.80475.dd

[47] Moinipour N , Barati M, Sahebkar A, Iranshahy M, Shakeri A. Protective effects of curcumin against iron-induced toxicity. Curr Pharm Biotechnol 2022;23:1020–7. 34521323 10.2174/1389201022666210914122846

[48] Kumari M , Rajak S, Singh SP, Murty US, Mahboob M, Grover P, Rahman MF. Biochemical alterations induced by acute oral doses of iron oxide nanoparticles in wistar rats. Drug Chem Toxicol 2013;36:296–305. 23025823 10.3109/01480545.2012.720988

[49] Radu Balas M , Din Popescu IM, Hermenean A, Cinteză OL, Burlacu R, Ardelean A, Dinischiotu A. Exposure to iron oxide nanoparticles coated with phospholipid-based polymeric micelles induces biochemical and histopathological pulmonary changes in mice. Int J Mol Sci 2015;16:29417–35. 26690409 10.3390/ijms161226173 PMC4691116

[50] Sadeghi L , Yousefi Babadi V, Espanani HR. Toxic effects of the Fe_2_O_3_ nanoparticles on the liver and lung tissue. Bratisl Lek Listy 2015;116:373–8. 26084739 10.4149/bll_2015_071

