# Supplementary material for: Comparative time-dependent proteomics reveal the tolerance of cancer cells to magnetic iron oxide nanoparticles
Source: Regen Biomater. 2024 Jun 4;11:rbae065. doi: 10.1093/rb/rbae065 (PMC11199825; doi:10.1093/rb/rbae065)
Supplement: rbae065_Supplementary_Data [file rbae065_supplementary_data.docx]

**Comparative time-dependent proteomics reveal the tolerance of cancer cells to magnetic iron oxide nanoparticles**

Supplementary Materials

**Table S1.** Characterization of synthesized Si-IONPs.

| **Characterization (Method)** | **Si-IONPs** |
| --- | --- |
| Particle size (TEM/SEM) | 93 ± 10 nm |
| Hydrodynamic size (DLS) | 190.5 ± 3.5 nm (470.9 ± 11.4 nm in medium) |
| Polydispersity index (DLS) | 0.125 (0.417 nm in medium) |
| Zeta potential (DLS) | −2.6 ± 0.3 mV (-9.82 ± 0.8 mV in medium) |
| Crystal structure (XRD) | Cubic inverse spinel (PDF 65-3107) |
| Surface modification (FTIR) | –NH_2_, Si–O |
| Saturation magnetization (VSM) | 87.96 emu/g |

TEM: transmission electron microscope; SEM: scanning electron microscope; DLS: dynamic light scattering; XRD: X-ray diffraction; FTIR: flourier transform infrared spectroscopy; VSM: vibrating sample magnetometer.

**Table S2.** Details for antibodies used in Western blotting.

| **Antibodies** | **Source** | **Dilution ratio** | **Supplier** |
| --- | --- | --- | --- |
| **HMOX1** | Rabbit | 1:1000 | Proteintech |
| **TRAP1** | Rabbit | 1:1000 | Abcam |
| **FTH1** | Rabbit | 1:1000 | Abcam |
| **β-actin** | Rabbit | 1:3000 | Proteintech |


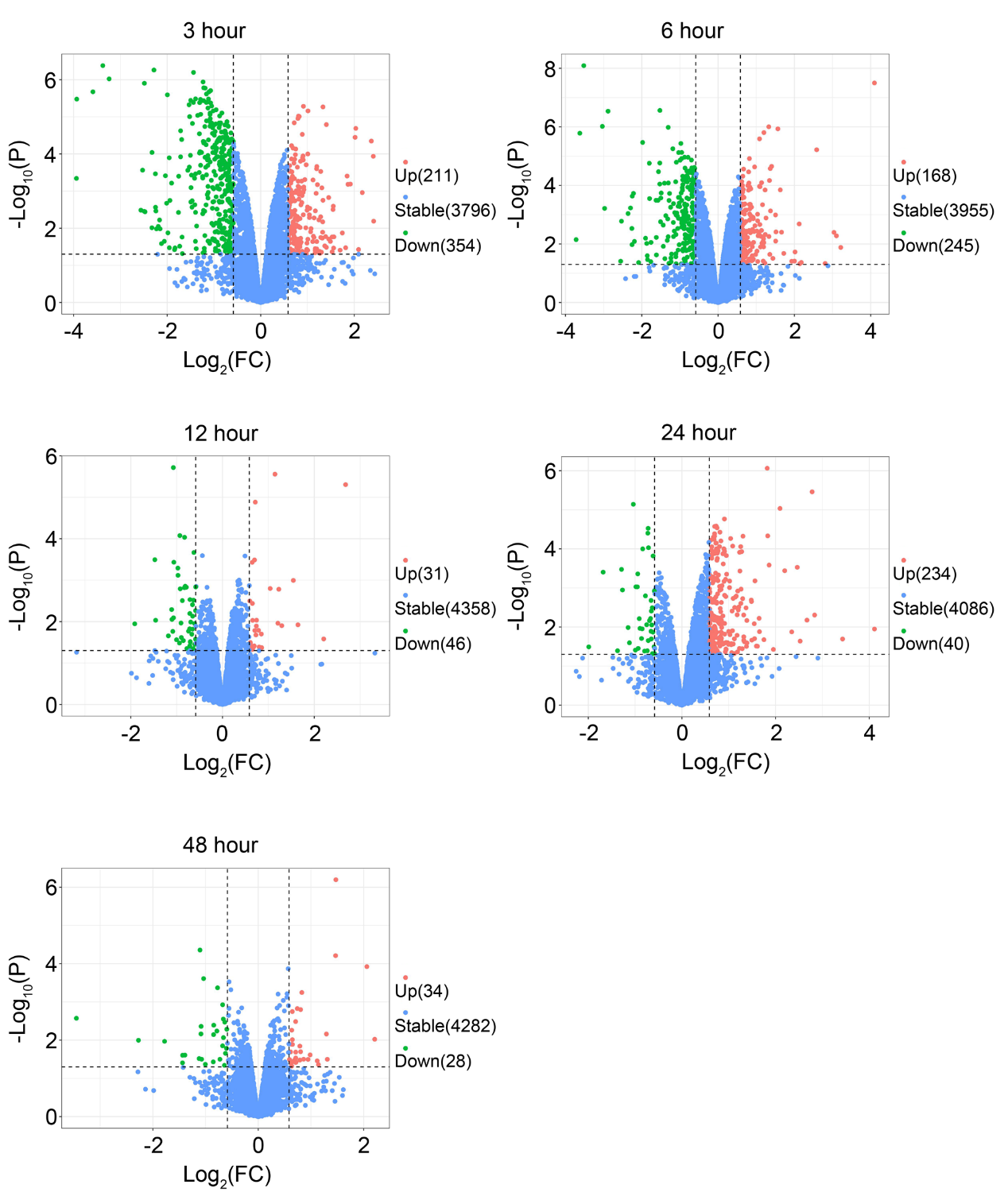


**Figure S1.** The volcano plots of DEPs of 293T cells at each timepoint.

**Table S3.** KEGG pathways enriched by DEPs of Hela and 293T.

| **Description** | **Hela** | **293t** |
| --- | --- | --- |
| **RNA degradation** | PFKL\|SKIC2\|**SKIC3**\|XRN2\|DIS3\|**MTREX**\|LSM1\|LSM3\|EXOSC3\|**EXOSC1**\|**DCP1A**\|EXOSC5\|EDC3\|**PNPT1** | CNOT2\|CNOT3\|EXOSC10\|**SKIC3**\|**MTREX**\|EDC4\|**EXOSC1**\|**DCP1A**\|SKIC8\|**PNPT1**\|DHX36 |
| **Apoptosis** | ATM\|BCL2\|**CASP8**\|**CTSC**\|**CTSB**\|CTSL\|CTSZ\|HRAS\|ITPR1\|LMNA\|**NFKBIA**\|PTPN13\|IKBKG\|**TNFRSF10B** | AKT2\|APAF1\|CASP2\|CASP7\|**CASP8**\|**CTSC**\|**CTSB**\|**NFKBIA**\|PIK3R1\|SPTAN1\|FADD\|**TNFRSF10B** |
| **Protein export** | OXA1L\|**SRP54**\|**SRP68**\|SRPRA\|**SEC61G** | SRP19\|**SRP54**\|**SRP68**\|**SEC61G**\|SEC11C |
| **Reactive oxygen species** | **ATP5F1D**\|BRAF\|CAT\|GSTT1\|**HMOX1**\|HRAS\|COX2\|**NDUFA2**\|NDUFA8\|NDUFAB1\|NDUFB4\|NDUFB10\|NDUFS1\|NDUFV1\|NDUFS4\|NDUFS8\|NDUFV2\|**NFKBIA**\|PTPRJ\|SOD2\|UQCRC1\|UQCRH\|**VDAC3**\|IKBKG\|UQCRQ\|**UQCR10**\|NDUFA13\|NDUFB11\|NDUFA11\|GSTT2B | ABL1\|AKT2\|**ATP5F1D**\|CYC1\|**HMOX1**\|MGST1\|CYTB\|**NDUFA2**\|NDUFA3\|**NFKBIA**\|PIK3R1\|VDAC2\|**VDAC3**\|COX7A2L\|PRKD3\|**UQCR10** |
| **Cell cycle** | ATM\|BUB1B\|CDC20\|CDK4\|**CDK6\|CDK7**\|MAD2L1\|MCM2\|MCM3\|MCM4\|MCM5\|MCM6\|MCM7\|ORC4\|PPP2R1B\|**PPP2R5E**\|PRKDC\|RAD21\|TTK\|MAD1L1\|**CCNB2**\|**NDC80**\|WAPL\|**PDS5A**\|ANAPC7 | ABL1\|**CDK6\|CDK7**\|SFN\|ORC5\|PPP2R5A\|**PPP2R5E**\|SKP2\|**CCNB2**\|RBX1\|ANAPC10\|**NDC80**\|STAG2\|**PDS5A**\|ANAPC4\|ANAPC1 |
| **mRNA surveillance pathway** | **CSTF3**\|**FUS**\|PPP1CC\|PPP2R1B\|**PPP2R5E**\|DDX39B\|**EIF4A3**\|PAPOLA\|**NUDT21**\|**CPSF6**\|NCBP2\|UPF2\|DAZAP1\|CPSF3\|**PYM1**\|MSI2 | CSTF1\|**CSTF3**\|**FUS**\|MSI1\|PPP2R5A\|**PPP2R5E**\|**EIF4A3**\|ALYREF\|**NUDT21**\|**CPSF6**\|PELO\|CPSF2\|**PYM1** |
| **Biosynthesis of amino acids** | ACY1\|ASL\|**CTH**\|PFKL\|PSPH\|ALDH18A1\|SHMT1\|GPT2 | ARG2\|**CTH**\|GLUL\|MTR\|PGK2\|PYCR1\|SHMT2\|PYCR2 |
| **mTOR signaling pathway** | BRAF\|DVL2\|HRAS\|RHEB\|**RPS6**\|TSC2\|EIF4E2\|**TELO2**\|**LAMTOR5**\|LAMTOR1\|**RRAGC**\|**SEH1L**\|FNIP1\|LAMTOR4\|CASTOR2 | AKT2\|ATP6V1E1\|EIF4EBP1\|PIK3R1\|**RPS6**\|SKP2\|**TELO2**\|**LAMTOR5**\|**RRAGC**\|MLST8\|**SEH1L** |
| **RIG-I-like receptor signaling pathway** | **CASP8**\|**DDX3X**\|**NFKBIA**\|**TRIM25**\|IKBKG\|ISG15\|**TKFC**\|TBK1\|NLRX1 | **CASP8**\|**DDX3X**\|**NFKBIA**\|MAP3K7\|**TRIM25**\|FADD\|**TKFC** |
| **Ferroptosis** | ACSL1\|**FTH1**\|**HMOX1**\|**TF**\|TFRC\|**VDAC3**\|LPCAT3\|SLC39A14 | **FTH1**\|**HMOX1**\|**TF**\|VDAC2\|**VDAC3** |
| **Lysosome** | **CTSC**\|AP3S1\|TPP1\|**CLTA**\|**CLTB**\|**CLTC**\|**CTSB**\|CTSL\|CTSZ\|**GAA**\|GUSB\|HEXA\|HEXB\|IGF2R\|**LAMP2**\|MAN2B1\|NPC1\|AP3D1\|ATP6V0D1\|PPT2\|GGA2\|GNPTG | ATP6AP1\|**CTSC**\|**CLTA**\|**CLTB**\|**CLTC**\|**CTSB**\|DMXL1\|DNASE2\|**GAA**\|GM2A\|**LAMP2**\|LGMN |
| **Endocytosis** | AP2A2\|CAV1\|AP2M1\|**CLTA**\|**CLTB**\|**CLTC**\|**DNM2**\|HRAS\|HSPA1B\|HSPA2\|**HSPA6**\|IGF2R\|LDLR\|TFRC\|RABEP1\|ARPC3\|ARFGEF1\|**RAB31**\|SPART\|SH3GLB2\|VPS25\|ARFGAP2\|AGAP3\|WIPF2 | BIN1\|**CLTA**\|**CLTB**\|**CLTC**\|**DNM2**\|HLA-A\|HLA-C\|HSPA1L\|**HSPA6**\|USP8\|VPS26A\|IST1\|**RAB31**\|WASHC4\|EHD4\|EHD2\|VPS29\|SH3GLB2\|SMAP1\|SMAP2 |
| **Synaptic vesicle cycle** | AP2A2\|AP2M1\|**CLTA**\|**CLTB**\|**CLTC**\|**DNM2**\|**SLC1A3**\|**STXBP1**\|ATP6V0D1\|RIMS1 | ATP6V1E1\|**CLTA**\|**CLTB**\|**CLTC**\|**DNM2**\|**SLC1A3**\|STX3\|**STXBP1** |
| **Peroxisome** | ABCD1\|CAT\|ACSL1\|HMGCL\|**PEX14**\|SOD2\|**PEX11B**\|ECI2\|PMVK\|DHRS4\|AMACR\|**HACL1**\|**FAR1** | **PEX14**\|PEX5\|PEX3\|**PEX11B**\|PEX16\|**HACL1**\|**FAR1** |
| **Nucleotide metabolism** | ADA\|ITPA\|NME3\|**RRM2**\|**TK1**\|**TYMS**\|UCK2\|ASMTL\|HDDC2\|NT5C3A\|GMPR2\|**UCKL1**\|CTPS2 | ENPP1\|**RRM2**\|**TK1**\|**TYMS**\|NT5C\|**UCKL1**\|UCK1\|NTPCR |
| **Pyrimidine metabolism** | NME3\|**RRM2**\|**TK1**\|**TYMS**\|UCK2\|ASMTL\|HDDC2\|NT5C3A\|**UCKL1**\|CTPS2 | NUDT2\|DHODH\|ENPP1\|**RRM2**\|**TK1**\|**TYMS**\|NT5C\|**UCKL1**\|UCK1 |
| **Autophagy - animal** | BCL2\|**CTSB**\|CTSL\|HRAS\|ITPR1\|**LAMP2**\|RHEB\|RRAS\|TSC2\|IRS2\|SQSTM1\|GABARAPL2\|TBK1\|PIK3R4\|**RRAGC**\|**MTMR14**\|RAB33B | AKT2\|**CTSB**\|**LAMP2**\|PIK3C3\|PIK3R1\|MAP3K7\|IRS4\|SQSTM1\|WDR41\|**RRAGC**\|MLST8\|**MTMR14** |
| **Antifolate resistance** | **DHFR**\|SHMT1\|**TYMS**\|IKBKG\|GGH\|ABCG2 | **DHFR**\|FPGS\|SHMT2\|**TYMS** |
| **One carbon pool by folate** | **DHFR**\|SHMT1\|**TYMS**\|**MTHFS** | **DHFR**\|MTR\|SHMT2\|**TYMS**\|**MTHFS** |
| **p53 signaling pathway** | ATM\|BCL2\|**CASP8**\|CDK4\|**CDK6**\|**RRM2**\|TSC2\|**TNFRSF10B**\|**CCNB2** | APAF1\|**CASP8**\|**CDK6**\|SFN\|**RRM2**\|**TNFRSF10B**\|**CCNB2** |
| **Thermogenesis** | ACTL6A\|ATP5F1D\|CPT2\|ACSL1\|HRAS\|COX2\|NDUFA2\|NDUFA8\|NDUFAB1\|NDUFB4\|NDUFB10\|NDUFS1\|NDUFV1\|NDUFS4\|NDUFS8\|NDUFV2\|MAP2K3\|RHEB\|RPS6\|SMARCA4\|SMARCD1\|SMARCD2\|TSC2\|UQCRC1\|UQCRH\|ARID1A\|UQCRQ\|COA3\|UQCR10\|NDUFA13\|NDUFAF1\|NDUFB11\|NDUFA11 | #N/A |
| **Retrograde endocannabinoid signaling** | ITPR1\|NDUFA2\|NDUFA8\|NDUFAB1\|NDUFB4\|NDUFB10\|NDUFS1\|NDUFV1\|NDUFS4\|NDUFS8\|NDUFV2\|RIMS1\|NDUFA13\|NDUFB11\|NDUFA11\|DAGLB | #N/A |
| **Nucleocytoplasmic transport** | KPNA1\|RANBP2\|DDX39B\|NUP214\|EIF4A3\|NUP153\|NCBP2\|UPF2\|NUP37\|NUP85\|SEH1L\|PYM1\|THOC3\|NUP43 | #N/A |
| **DNA replication** | MCM2\|MCM3\|MCM4\|MCM5\|MCM6\|MCM7\|POLD1\|POLD2\|RFC3\|RPA1\|RPA3\|POLE4 | #N/A |
| **Steroid biosynthesis** | CYP51A1\|DHCR7\|SOAT1\|HSD17B7 | #N/A |
| **Fatty acid metabolism** | ACADS\|ACADVL\|CPT2\|ACSL1\|HADH\|HSD17B8\|PPT2\|TECR\|MECR\|CBR4 | #N/A |
| **Fatty acid degradation** | ACADS\|ACADVL\|ALDH1B1\|CPT2\|ACSL1\|HADH\|ECI2 | #N/A |
| **Endocrine and other factor-regulated calcium reabsorption** | AP2A2\|ATP2B4\|AP2M1\|CLTA\|CLTB\|CLTC\|DNM2 | #N/A |
| **Bacterial invasion of epithelial cells** | CAV1\|CLTA\|CLTB\|CLTC\|DNM2\|SEPTIN2\|SHC1\|BCAR1\|ARPC3\|SEPTIN9\|SEPTIN6\|SEPTIN8\|SEPTIN11 | #N/A |
| **Biosynthesis of cofactors** | ALDH1B1\|DHFR\|GUSB\|HPD\|MOCS2\|NME3\|PPOX\|SHMT1\|UROD\|GGH\|QPRT\|GMPPB\|GMPPA\|COQ6\|PNPO\|CTPS2\|FLAD1\|NAPRT\|EARS2\|MMAB | #N/A |
| **Aminoacyl-tRNA biosynthesis** | CARS1\|FARSA\|FARSB\|FARS2\|YARS2\|AARS2\|NARS2\|TARS2\|EARS2\|GATC | #N/A |
| **Drug metabolism - other enzymes** | GSTT1\|GUSB\|ITPA\|NME3\|RRM2\|TK1\|TPMT\|UCK2\|UCKL1\|GSTT2B | #N/A |
| **Mismatch repair** | PMS2\|POLD1\|POLD2\|RFC3\|RPA1\|RPA3 | #N/A |
| **Nucleotide excision repair** | CDK7\|POLD1\|POLD2\|RFC3\|RPA1\|RPA3\|POLE4 | #N/A |
| **Homologous recombination** | ATM\|MRE11\|POLD1\|POLD2\|RPA1\|RPA3\|RAD50 | #N/A |
| **Cholesterol metabolism** | APOA1\|APOB\|APOC3\|APOE\|LDLR\|NPC1\|PLTP\|SOAT1\|VDAC3 | #N/A |
| **Galactose metabolism** | AKR1B1\|GAA\|GALE\|GALK1\|PFKL | #N/A |
| **NF-kappa B signaling pathway** | ATM\|BCL2\|ICAM1\|NFKB2\|NFKBIA\|PLCG1\|PLCG2\|TRIM25\|IKBKG\|MALT1 | #N/A |
| **Cytosolic DNA-sensing pathway** | CASP8\|GSDME\|NFKBIA\|IKBKG\|POLR3F\|POLR3C\|TBK1\|POLR3E | #N/A |
| **HIF-1 signaling pathway** | BCL2\|HMOX1\|PFKL\|PLCG1\|PLCG2\|RPS6\|TF\|TFRC\|MKNK1\|EIF4E2\|EGLN1 | #N/A |
| **Basal transcription factors** | CDK7\|GTF2F1\|GTF2F2\|TAF7\|TAF10\|TBPL1 | #N/A |
| **Valine, leucine and isoleucine degradation** | ACADS\|ALDH1B1\|BCKDHB\|HADH\|HMGCL\|HMGCS1\|IVD\|MMUT\|PCCB\|MCCC2 | #N/A |
| **Amino sugar and nucleotide sugar metabolism** | GALE\|GALK1\|HEXA\|HEXB\|GMPPB\|GMPPA\|AMDHD2\|CYB5R1\|FCSK | #N/A |
| **Human T-cell leukemia virus 1 infection** | ATM\|BUB1B\|CDC20\|CDK4\|HRAS\|ICAM1\|MAD2L1\|NFKB2\|NFKBIA\|VDAC3\|TRRAP\|MAD1L1\|IKBKG\|CCNB2\|TBPL1\|ANAPC7\|CRTC3 | #N/A |
| **Porphyrin metabolism** | GUSB\|HMOX1\|PPOX\|UROD\|EARS2\|MMAB | #N/A |
| **Fructose and mannose metabolism** | AKR1B1\|PFKL\|SORD\|TKFC\|GMPPB\|GMPPA\|FCSK | #N/A |
| **Insulin signaling pathway** | BRAF\|GYS1\|HRAS\|PCK2\|PHKA2\|PHKG2\|PPP1CC\|PYGB\|RHEB\|RPS6\|SHC1\|TSC2\|MKNK1\|IRS2\|EIF4E2 | #N/A |
| **Hepatocellular carcinoma** | ACTL6A\|BRAF\|CDK4\|CDK6\|DVL2\|GSTT1\|HMOX1\|HRAS\|PLCG1\|PLCG2\|SHC1\|SMARCA4\|SMARCD1\|SMARCD2\|ARID1A\|AXIN1\|GSTT2B | #N/A |
| **Butanoate metabolism** | ACADS\|HADH\|HMGCL\|HMGCS1\|L2HGDH | #N/A |
| **Various types of N-glycan biosynthesis** | HEXA\|HEXB\|STT3A\|MAN2A1\|ALG3\|MAN1B1\|ALG1 | #N/A |
| **Protein processing in endoplasmic reticulum** | BAG1\|BCL2\|HSPA1B\|HSPA2\|HSPA6\|STT3A\|RRBP1\|MOGS\|SEC23B\|SEC24A\|OS9\|MAN1B1\|SEC31A\|SEC61G\|YOD1\|SIL1\|DNAJC5 | #N/A |
| **Cellular senescence** | ATM\|CDK4\|CDK6\|HRAS\|ITPR1\|MRE11\|PPP1CC\|MAP2K3\|MAP2K6\|RHEB\|RRAS\|TSC2\|VDAC3\|SQSTM1\|CCNB2\|RAD50 | #N/A |
| **Other types of O-glycan biosynthesis** | GALNT1\|GALNT2\|GALNT6\|POFUT2\|XXYLT1\|EOGT | #N/A |
| **Focal adhesion** | ACTN4\|ACTN1\|ARHGAP5\|BCL2\|BRAF\|CAV1\|FLNA\|FLNB\|FLNC\|HRAS\|PPP1R12A\|PPP1CC\|RAP1B\|SHC1\|VASP\|VTN\|BCAR1 | #N/A |
| **Proteoglycans in cancer** | BRAF\|CAV1\|CTSL\|DDX5\|FLNA\|FLNB\|FLNC\|HRAS\|ITPR1\|PPP1R12A\|PLCG1\|PLCG2\|PPP1CC\|RPS6\|RRAS\|VTN\|PDCD4 | #N/A |
| **Glyoxylate and dicarboxylate metabolism** | CAT\|MMUT\|PCCB\|SHMT1\|GRHPR | #N/A |
| **Propanoate metabolism** | ACADS\|BCKDHB\|MMUT\|PCCB\|SUCLG2 | #N/A |
| **Sulfur relay system** | MOCS2\|MPST\|URM1 | #N/A |
| **Sulfur metabolism** | MPST\|SELENBP1\|PAPSS1 | #N/A |
| **Fatty acid biosynthesis** | ACSL1\|HSD17B8\|MECR\|CBR4 | #N/A |
| **Ubiquitin mediated proteolysis** | #N/A | CDC34\|SKP2\|UBE2A\|UBE2E1\|UBE2G1\|HERC1\|RBX1\|ANAPC10\|UBE2C\|MGRN1\|UBE2S\|ANAPC4\|ANAPC1 |
| **NOD-like receptor signaling pathway** | #N/A | CASP8\|CTSB\|NFKBIA\|PKN1\|RNASEL\|MAP3K7\|VDAC2\|VDAC3\|FADD\|MFN2\|TAB1\|MFN1 |
| **Toll-like receptor signaling pathway** | #N/A | AKT2\|CASP8\|NFKBIA\|PIK3R1\|MAP3K7\|FADD\|TAB1\|TOLLIP |
| **Arginine and proline metabolism** | #N/A | ARG2\|GAMT\|PYCR1\|P4HA2\|PYCR2\|LAP3 |
| **N-Glycan biosynthesis** | #N/A | B4GALT1\|MAN2A1\|MGAT2\|MOGS\|ALG5\|ALG1 |
| **Antigen processing and presentation** | #N/A | CTSB\|HLA-A\|HLA-C\|HSPA1L\|HSPA6\|LGMN\|RFXAP |

#N/A: no DEPs were identified of quantified on this pathway.

**Table S4.** GO pathways enriched by DEPs of Hela and 293T.

| **Description** | **Hela** | **293T** |
| --- | --- | --- |
| **Apoptotic signaling pathway** | ATM\|BCL2\|**CASP8**\|CAV1\|CD70\|**DDX3X**\|DDX5\|**HMOX1**\|HRAS\|ITPR1\|PAWR\|PRKDC\|DPF2\|**SCN2A**\|SOD2\|**TNFRSF10B**\|PPM1F\|**MYBBP1A**\|DIDO1\|**FIS1**\|NDUFA13\|QRICH1\|USP47 | ABL1\|ANXA6\|APAF1\|CASP2\|**CASP8**\|**DDX3X**\|SFN\|GPX1\|**HMOX1**\|PIK3R1\|**SCN2A**\|VDAC2\|STK24\|FADD\|**TNFRSF10B**\|PDCD6\|**MYBBP1A**\|**FIS1** |
| **Autophagy of peroxisome** | ATM\|**SQSTM1**\|PIK3R4 | PIK3C3\|PEX5\|**SQSTM1** |
| **Cellular response to environmental stimulus** | AKR1B1\|**RHOB**\|ATM\|**CASP8**\|**DAG1**\|**DDX3X**\|**DNM2**\|HRAS\|**NPM1**\|POLD1\|**RPL26**\|**SCN2A**\|**TNFRSF10B**\|ARHGEF2\|PIEZO1\|ZMPSTE24\|NFAT5\|**SLC38A2**\|USP47\|PBK\|FIGNL1\|ACTR5 | AKT2\|**RHOB**\|ATP1A2\|CASP2\|**CASP8**\|**DAG1**\|**DDX3X**\|**DNM2**\|ECT2\|HSF1\|MAG\|**NPM1**\|PIK3R1\|PLEC\|**RPL26**\|**SCN2A**\|FADD\|**TNFRSF10B**\|RBX1\|INTS7\|**SLC38A2**\|MLST8\|TNKS1BP1\|DHX36 |
| **Cellular response to oxidative stress** | **RHOB**\|ATM\|BCL2\|CAT\|**HMOX1**\|HSPA1B\|**PEX14**\|RPS3\|SOD2\|STAT6\|**SQSTM1**\|ANKRD2\|SLC25A24\|**CHCHD2**\|**ERMP1**\|**PNPT1**\|AGAP3 | ABL1\|NUDT2\|**RHOB**\|ECT2\|GPX1\|**HMOX1**\|HSF1\|MAPT\|MGST1\|**PEX14**\|PEX5\|PYCR1\|RPS3\|STK24\|PRKRA\|**SQSTM1**\|CCS\|SETX\|PYCR2\|**CHCHD2**\|PYROXD1\|**ERMP1**\|**PNPT1** |
| **Energy derivation by oxidation of organic compounds** | ACADVL\|AGL\|**ATP5F1D**\|CAT\|**GAA**\|GYG1\|GYS1\|IL6ST\|COX2\|**NDUFA2**\|NDUFA8\|NDUFAB1\|NDUFB4\|NDUFB10\|NDUFS1\|NDUFV1\|NDUFS4\|NDUFS8\|NDUFV2\|OXA1L\|PHKA2\|PHKG2\|PPP1CC\|PYGB\|SOD2\|UQCRC1\|UQCRH\|SUCLG2\|**MYBBP1A**\|**POLG2**\|UQCRQ\|**UQCR10**\|NDUFA13\|NDUFAF1\|NDUFB11\|DHTKD1\|COQ9\|NDUFA11\|SDHAF4 | AKT2\|**ATP5F1D**\|CYC1\|**GAA**\|GBE1\|CYTB\|**NDUFA2**\|NDUFA3\|OGDH\|PLEC\|PYGM\|SLC25A12\|COX7A2L\|**MYBBP1A**\|**POLG2**\|**UQCR10** |
| **Intracellular iron ion homeostasis** | ABCB7\|**FTH1**\|**HMOX1**\|**TF**\|TFRC\|PICALM\|ATP6V0D1\|ISCU\|SLC39A14\|FLVCR1\|EGLN1\|NDFIP1 | ATP6AP1\|**FTH1**\|**HMOX1**\|HPX\|**TF**\|ABCB6\|BDH2 |
| **Intrinsic Apoptotic signaling pathway** | ATM\|BCL2\|**DDX3X**\|DDX5\|**HMOX1**\|HRAS\|ITPR1\|PRKDC\|**SCN2A**\|SOD2\|**TNFRSF10B**\|PPM1F\|**MYBBP1A**\|QRICH1\|USP47 | ABL1\|APAF1\|CASP2\|**DDX3X**\|SFN\|GPX1\|**HMOX1**\|PIK3R1\|**SCN2A**\|STK24\|**TNFRSF10B**\|**MYBBP1A** |
| **Iron ion transport** | ABCB7\|**CLTC**\|**DNM2**\|**FTH1**\|**TF**\|TFRC\|SLC39A14\|FLVCR1 | **CLTC**\|**DNM2**\|**FTH1**\|HPX\|**TF**\|ABCB6 |
| **Positive regulation of apoptotic process** | RHOB\|ATM\|BCL2\|**CASP8**\|CAV1\|**CTSC**\|CTSL\|**DDX3X**\|GSDME\|**DNM2**\|EIF5A\|**GRN**\|NR3C1\|**HMOX1**\|IGF2R\|PAWR\|PLCG1\|PPP2R1B\|PRKDC\|MAP2K6\|**RPL11**\|**RPL26**\|**RPS3**\|**RPS6**\|**RPS7**\|SOD2\|**TNFRSF10B**\|ARHGEF7\|**SQSTM1**\|RACK1\|**MYBBP1A**\|FAF1\|MTCH2\|**PDCD4**\|**FIS1**\|QRICH1\|**CCAR1**\|CAMK1D\|PLEKHF1\|FNIP1 | ABL1\|BIN1\|APAF1\|APBB1\|RHOB\|CASP2\|**CASP8**\|CDC34\|**CTSC**\|**DDX3X**\|**DNM2**\|ECT2\|B4GALT1\|**GRN**\|HTT\|**HMOX1**\|**RPL11**\|**RPL26**\|**RPS3**\|**RPS6**\|**RPS7**\|PRKRA\|FADD\|**TNFRSF10B**\|**SQSTM1**\|MAGED1\|MFN2\|**MYBBP1A**\|DDX20\|**PDCD4**\|**FIS1**\|**CCAR1** |
| **Positive regulation of apoptotic signaling pathway** | CAV1\|**CTSC**\|CTSL\|GSDME\|EIF5A\|PPP2R1B\|**RPL11**\|**RPL26**\|**RPS3**\|**RPS7**\|RACK1\|FAF1\|**FIS1**\|PLEKHF1 | APAF1\|CASP2\|**CTSC**\|**RPL11**\|**RPL26**\|**RPS3**\|**RPS7**\|PRKRA\|FADD\|MAGED1\|**FIS1** |
| **Positive regulation of cell death** | **RHOB**\|ATM\|BCL2\|**CASP8**\|CAV1\|**CTSC**\|CTSL\|**DDX3X**\|GSDME\|**DNM2**\|EIF5A\|**GRN**\|NR3C1\|**HMOX1**\|IGF2R\|COX2\|PAWR\|PLCG1\|PPP2R1B\|PRKDC\|MAP2K6\|**RPL11**\|**RPL26**\|**RPS3**\|**RPS6**\|**RPS7**\|SOD2\|PICALM\|**TNFRSF10B**\|ARHGEF7\|**SQSTM1**\|KATNB1\|RACK1\|**MYBBP1A**\|FAF1\|HEBP2\|MTCH2\|**PDCD4**\|**FIS1**\|QRICH1\|**CCAR1**\|CAMK1D\|PLEKHF1\|FNIP1 | ABL1\|BIN1\|APAF1\|APBB1\|**RHOB**\|CASP2\|**CASP8**\|CDC34\|**CTSC**\|**DDX3X**\|**DNM2**\|ECT2\|B4GALT1\|**GRN**\|HTT\|**HMOX1**\|MAPT\|PHB1\|**RPL11**\|**RPL26**\|**RPS3**\|**RPS6**\|**RPS7**\|PRKRA\|FADD\|**TNFRSF10B**\|**SQSTM1**\|MAGED1\|MFN2\|**MYBBP1A**\|HTATIP2\|DDX20\|**PDCD4**\|**FIS1**\|**CCAR1** |
| **Negative regulation of cysteine-type endopeptidase activity involved in apoptotic process** | **DDX3X**\|MAGEA3\|NOL3\|**DNAJB6**\|**LAMTOR5**\|**PIH1D1**\|USP47\|FNIP1 | **DDX3X**\|SFN\|GPX1\|**DNAJB6**\|**LAMTOR5**\|NLE1\|**PIH1D1** |
| **Positive regulation of intrinsic apoptotic signaling pathway** | CAV1\|GSDME\|EIF5A\|**RPL11**\|**RPL26**\|**RPS3**\|**RPS7**\|RACK1\|**FIS1**\|PLEKHF1 | **RPL11**\|**RPL26**\|**RPS3**\|**RPS7**\|PRKRA\|**FIS1** |
| **Positive regulation of intrinsic apoptotic signaling pathway by p53 class mediator** | EIF5A\|**RPL11**\|**RPL26**\|**RPS7** | **RPL11**\|**RPL26**\|**RPS7** |
| **Clathrin-dependent endocytosis** | AP2A2\|AP2M1\|**CLTA**\|**CLTB**\|**CLTC**\|**GAK**\|ITSN1\|PICALM\|FNBP1L | **CLTA**\|**CLTB**\|**CLTC**\|**GAK**\|FCHO2 |
| **Regulation of apoptotic signaling pathway** | BCL2\|CAV1\|**CTSC**\|**CTH**\|CTSL\|**DDX3X**\|GSDME\|EIF5A\|**HMOX1**\|**HNRNPK**\|HSPA1B\|ICAM1\|LMNA\|MAGEA3\|MIF\|PPP2R1B\|PSMD10\|**RPL11**\|**RPL26**\|**RPS3**\|**RPS7**\|SOD2\|TPT1\|URI1\|NOL3\|ARHGEF2\|BAG5\|RACK1\|FAF1\|**FIS1**\|NDUFA13\|**PIH1D1**\|USP47\|FIGNL1\|PLEKHF1\|**PTPMT1** | APAF1\|CASP2\|**CTSC**\|**CTH**\|**DDX3X**\|GPX1\|HTT\|**HMOX1**\|**HNRNPK**\|PF4\|**RPL11**\|**RPL26**\|**RPS3**\|**RPS7**\|VDAC2\|PRKRA\|FADD\|MAGED1\|**FIS1**\|NLE1\|**PIH1D1**\|**PTPMT1** |
| **Regulation of cysteine-type endopeptidase activity involved in apoptotic process** | **CASP8**\|**DDX3X**\|MAGEA3\|ROBO1\|**RPS3**\|NOL3\|PPM1F\|**DNAJB6**\|RACK1\|**LAMTOR5**\|MALT1\|**FIS1**\|NDUFA13\|**PIH1D1**\|USP47\|FNIP1 | APAF1\|CASP2\|**CASP8**\|**DDX3X**\|SFN\|GPX1\|HSF1\|MAPT\|**RPS3**\|PDCD6\|**DNAJB6**\|**LAMTOR5**\|**FIS1**\|NLE1\|**PIH1D1**\|MUL1 |
| **Regulation of intrinsic apoptotic signaling pathway** | BCL2\|CAV1\|**DDX3X**\|GSDME\|EIF5A\|**HNRNPK**\|MAGEA3\|MIF\|**RPL11**\|**RPL26**\|**RPS3**\|**RPS7**\|SOD2\|TPT1\|URI1\|NOL3\|ARHGEF2\|BAG5\|RACK1\|**FIS1**\|NDUFA13\|USP47\|FIGNL1\|PLEKHF1\|**PTPMT1** | **DDX3X**\|GPX1\|**HNRNPK**\|**RPL11**\|**RPL26**\|**RPS3**\|**RPS7**\|VDAC2\|PRKRA\|**FIS1**\|**PTPMT1** |
| **Response to oxidative stress** | APOE\|APP\|**RHOB**\|ATM\|ATOX1\|BCL2\|CAT\|**HMOX1**\|HSPA1B\|**KRT1**\|NDUFB4\|NDUFS8\|**PEX14**\|**RPS3**\|SOD2\|SP1\|STAT6\|**TRIM25**\|PXDN\|**SQSTM1**\|PDLIM1\|ANKRD2\|SLC25A24\|**CHCHD2**\|ERMP1\|**PNPT1**\|NAPRT\|AGAP3 | ABL1\|NUDT2\|**RHOB**\|ECT2\|GPX1\|**HMOX1**\|HSF1\|**KRT1**\|MAPT\|MGST1\|**PEX14**\|PEX5\|PYCR1\|**RPS3**\|**TRIM25**\|STK24\|PRKRA\|**SQSTM1**\|CCS\|SETX\|PYCR2\|**CHCHD2**\|PYROXD1\|ERMP1\|**PNPT1** |
| **Cellular respiration** | **ATP5F1D**\|CAT\|COX2\|**NDUFA2**\|NDUFA8\|NDUFAB1\|NDUFB4\|NDUFB10\|NDUFS1\|NDUFV1\|NDUFS4\|NDUFS8\|NDUFV2\|OXA1L\|SOD2\|UQCRC1\|UQCRH\|SUCLG2\|**MYBBP1A**\|**POLG2**\|UQCRQ\|**UQCR10**\|NDUFA13\|NDUFAF1\|NDUFB11\|DHTKD1\|COQ9\|NDUFA11\|SDHAF4 | **ATP5F1D**\|CYC1\|CYTB\|**NDUFA2**\|NDUFA3\|OGDH\|PLEC\|SLC25A12\|COX7A2L\|**MYBBP1A**\|**POLG2**\|**UQCR10** |
| **DNA repair** | ACTL6A\|ATM\|**CDC5L**\|**CDK7**\|CDK9\|CHD4\|**DDX1**\|MCM2\|MCM3\|MCM4\|MCM5\|MCM6\|MCM7\|MRE11\|MSH4\|**NPM1**\|**PMS2**\|POLD1\|**POLD2**\|PRKDC\|RAD21\|RECQL\|RFC3\|RPA1\|RPA3\|**RPS3**\|UBE2V2\|RNF113A\|**CHAF1B**\|TRRAP\|**SMARCA5**\|TRIP12\|RECQL4\|**MORF4L2**\|**PCLAF**\|RAD50\|ZMPSTE24\|**ASCC3**\|**PDS5A**\|SMCHD1\|**UBE2T**\|TEX264\|**TDP2**\|**GINS2**\|RTEL1\|**HPF1**\|USP47\|FANCI\|DMAP1\|RAD18\|**WDR48**\|ACTR5\|**HDGFL2**\|**ACTR8**\|**PAXX** | ABL1\|**CDC5L**\|**CDK7**\|**DDX1**\|HSF1\|TONSL\|**NPM1**\|**PMS2**\|**POLD2**\|**RPS3**\|UBE2A\|**CHAF1B**\|**SMARCA5**\|PARG\|TIMELESS\|USP10\|MTA1\|**MORF4L2**\|**PCLAF**\|RBX1\|**ASCC3**\|WDHD1\|SETX\|**PDS5A**\|**UBE2T**\|ASCC1\|**TDP2**\|**GINS2**\|**HPF1**\|**WDR48**\|CLSPN\|MMS19\|**HDGFL2**\|TNKS1BP1\|MGME1\|**ACTR8**\|**PAXX** |
| **Endocytosis** | AP2A2\|**AHSG**\|APOE\|APP\|CAV1\|AP2M1\|AP3S1\|**CLTA**\|**CLTB**\|**CLTC**\|CTBP1\|CTSL\|**DNM2**\|**GAK**\|HRAS\|IGF2R\|LDLR\|NPC1\|PI4KB\|ITSN1\|SNCG\|SURF4\|TFRC\|TSC2\|**VLDLR**\|VTN\|PICALM\|AP3D1\|RABEP1\|**CLINT1**\|**SNX17**\|RABEPK\|**RAB31**\|**FKBP15**\|DNAJC13\|CORO1C\|**HEATR5A**\|PACSIN3\|CD320\|FNBP1L\|SCYL2\|RABEP2\|RAB34\|REPS1\|MIA3 | ABL1\|**AHSG**\|BIN1\|ANXA3\|**CLTA**\|**CLTB**\|**CLTC**\|**DNM2**\|**GAK**\|MYO6\|PAK1\|ENPP1\|PIK3C3\|SYP\|**VLDLR**\|SCAMP1\|**CLINT1**\|**SNX17**\|**RAB31**\|USP33\|**FKBP15**\|**HEATR5A**\|RABGEF1\|EHD4\|EHD2\|FCHO2 |
| **Exocytosis** | BRAF\|RAB13\|RAP1B\|ITSN1\|**STXBP1**\|RABEPK\|ARFGEF1\|EXOC5\|**RAB31**\|RIMS1\|EXOC6B\|SDF4\|VPS18\|DNAJC5\|**TPRG1L**\|EXOC8\|MIA3 | ANXA3\|PAK1\|STX3\|**STXBP1**\|STXBP2\|SYNGR2\|SCAMP1\|RAB3D\|**RAB31**\|EXOC3\|EXOC7\|RABGEF1\|TRAPPC11\|**TPRG1L** |
| **Monoatomic ion homeostasis** | ABCB7\|APOE\|APP\|ATOX1\|ATP2B4\|BCL2\|CAV1\|FTH1\|GRN\|HEXB\|HMOX1\|ITPR1\|SCO1\|SLC1A3\|SLC12A4\|SOD2\|TF\|TFRC\|TPT1\|PICALM\|ATP6V0D1\|ISCU\|SLC39A14\|FLVCR1\|FIS1\|TMCO1\|EGLN1\|CLN6\|RMDN3\|TMEM165\|ATP13A1\|THADA\|MCUR1\|VPS33A\|NDFIP1\|PDZD8 | #N/A |
| **Cell population proliferation** | ADA\|APOA1\|BCL2\|BYSL\|CAV1\|CD70\|CDK9\|DVL2\|HMOX1\|HRAS\|KPNA1\|MCM7\|MRE11\|NFIB\|MED1\|RAP1B\|RPS6\|STAT6\|IRS2\|SQSTM1\|EIF2S2\|USP13\|ARHGEF2\|GINS1\|ARIH2\|TACC3\|IGF2BP1\|MALT1\|ASCC3\|BOP1\|LARP1\|PES1\|GNL3\|DAZAP1\|TBK1\|SBDS\|FERMT1\|FIGNL1\|IFT74\|NDFIP1\|FAM83D\|DAGLB | #N/A |
| **Positive regulation of cell cycle G1/S phase transition** | DDX3X\|PLCG2\|RRM2\|ADAM17\|ANKRD17\|CPSF3\|PAF1\|CDC73\|FAM83D | #N/A |
| **Cell cycle DNA replication** | MCM2\|MCM3\|MCM4\|MCM6\|TK1\|GINS1\|RTEL1\|GINS3 | #N/A |
| **Positive regulation of cell cycle G2/M phase transition** | APP\|CDK4\|HSPA2\|NPM1\|RCC2 | #N/A |
| **Inorganic ion homeostasis** | ABCB7\|APOE\|APP\|ATOX1\|ATP2B4\|BCL2\|CAV1\|FTH1\|HEXB\|HMOX1\|ITPR1\|SCO1\|SLC1A3\|SLC12A4\|SOD2\|TF\|TFRC\|TPT1\|PICALM\|ATP6V0D1\|ISCU\|SLC39A14\|FLVCR1\|FIS1\|TMCO1\|EGLN1\|RMDN3\|TMEM165\|ATP13A1\|THADA\|MCUR1\|NDFIP1\|PDZD8 | #N/A |
| **Lysosomal protein catabolic process** | TPP1\|LAMP2\|DPP7 | #N/A |
| **Lysosomal transport** | RHOB\|GAK\|GRN\|IGF2R\|LAMP2\|NPC1\|AP3D1\|GCC2\|PIK3R4\|VPS53\|SCYL2\|VPS18\|AKTIP\|VPS33A\|PLEKHF1\|GNPTG | #N/A |
| **Lysosome organization** | TPP1\|GAA\|GRN\|HEXA\|HEXB\|PI4KB\|CLN6\|LAMTOR1\|TMEM165\|VPS18\|AKTIP\|VPS33A\|RAB34 | #N/A |
| **Regulation of intrinsic apoptotic signaling pathway by p53 class mediator** | BCL2\|EIF5A\|HNRNPK\|MIF\|RPL11\|RPL26\|RPS7 | #N/A |
| **Regulation of intrinsic apoptotic signaling pathway in response to DNA damage** | BCL2\|HNRNPK\|MIF\|RPL26\|RPS3\|TPT1\|USP47 | #N/A |
| **Oxidative phosphorylation** | ATP5F1D\|COX2\|NDUFA2\|NDUFA8\|NDUFAB1\|NDUFB4\|NDUFB10\|NDUFS1\|NDUFV1\|NDUFS4\|NDUFS8\|NDUFV2\|UQCRC1\|UQCRH\|UQCRQ\|UQCR10\|NDUFA13\|NDUFAF1\|NDUFB11\|COQ9\|NDUFA11 | #N/A |
| **Regulation of intrinsic apoptotic signaling pathway in response to DNA damage by p53 class mediator** | BCL2\|HNRNPK\|MIF\|RPL26 | #N/A |
| **Regulation of nitric oxide biosynthetic process** | ASL\|ATP2B4\|CAV1\|DNM2\|PTX3\|SOD2\|DDAH2 | #N/A |
| **Negative regulation of intrinsic apoptotic signaling pathway** | BCL2\|DDX3X\|MAGEA3\|MIF\|SOD2\|TPT1\|URI1\|NOL3\|ARHGEF2\|BAG5\|NDUFA13\|USP47\|FIGNL1 | #N/A |
| **Lipid oxidation** | ACADS\|ACADVL\|ABCD1\|CPT2\|DECR1\|HADH\|IVD\|ECI2\|ILVBL\|AMACR\|HACL1 | #N/A |
| **Negative regulation of apoptotic signaling pathway** | BCL2\|CTH\|DDX3X\|HMOX1\|HSPA1B\|ICAM1\|LMNA\|MAGEA3\|MIF\|PSMD10\|SOD2\|TPT1\|URI1\|NOL3\|ARHGEF2\|BAG5\|NDUFA13\|PIH1D1\|USP47\|FIGNL1 | #N/A |
| **Fatty acid beta-oxidation** | ACADS\|ACADVL\|ABCD1\|CPT2\|DECR1\|HADH\|IVD\|ECI2\|AMACR | #N/A |
| **Fatty acid oxidation** | ACADS\|ACADVL\|ABCD1\|CPT2\|DECR1\|HADH\|IVD\|ECI2\|ILVBL\|AMACR\|HACL1 | #N/A |
| **Transferrin transport** | CLTC\|DNM2\|TFRC | #N/A |
| **ATP metabolic process** | ATP5F1D\|HSPA1B\|NDUFA2\|NDUFA8\|NDUFAB1\|NDUFB4\|NDUFB10\|NDUFS1\|NDUFV1\|NDUFS4\|NDUFS8\|NDUFV2\|NDUFA13\|NDUFB11\|FIGNL1\|ATP5MK\|NDUFA11 | #N/A |
| **Cellular homeostasis** | ABCB7\|APOE\|APP\|ATOX1\|ATP2B4\|BCL2\|CAV1\|DMD\|FTH1\|GAA\|GLS\|GRN\|HEXB\|HMOX1\|FOXK2\|ITPR1\|LAMP2\|PCK2\|RBM4\|SCO1\|SLC1A3\|SLC12A4\|SOD2\|TF\|TFRC\|TPT1\|PICALM\|IRS2\|ATP6V0D1\|RACK1\|UBE2C\|ISCU\|SLC39A14\|UBE2S\|FLVCR1\|FIS1\|TMCO1\|EGLN1\|CLN6\|RMDN3\|TMEM165\|ATP13A1\|THADA\|MCUR1\|VPS33A\|CCDC51\|NDFIP1\|PDZD8 | #N/A |
| **Cellular lipid catabolic process** | ACADS\|ACADVL\|ABCD1\|APOB\|APOC3\|CPT2\|DECR1\|HADH\|HEXA\|HEXB\|IVD\|PCCB\|PCK2\|PLCG1\|PLCG2\|ECI2\|ILVBL\|AMACR\|HACL1\|PLBD2\|DAGLB | #N/A |
| **Extrinsic apoptotic signaling pathway via death domain receptors** | #N/A | CASP8\|DDX3X\|PIK3R1\|FADD\|TNFRSF10B |
| **Peroxisome organization** | #N/A | PEX14\|PEX5\|PEX3\|PEX11B\|PEX16\|HACL1\|FIS1 |
| **Positive regulation of cysteine-type endopeptidase activity involved in apoptotic process** | #N/A | APAF1\|CASP2\|CASP8\|DDX3X\|HSF1\|MAPT\|RPS3\|PDCD6\|FIS1\|MUL1 |
| **Protein oxidation** | #N/A | APOA1\|GPX1\|CHCHD4 |
| **Protein targeting to peroxisome** | #N/A | PEX14\|PEX5\|PEX3\|PEX16\|HACL1 |
| **Response to hydrogen peroxide** | #N/A | ABL1\|RHOB\|ECT2\|GPX1\|HMOX1\|HSF1\|RPS3\|STK24\|SETX |
| **TRAIL-activated apoptotic signaling pathway** | #N/A | CASP8\|FADD\|TNFRSF10B |
| **Cellular response to nutrient levels** | #N/A | PLIN2\|ALB\|GLUL\|HMOX1\|HNRNPL\|LAMP2\|PIK3C3\|STK24\|MYBBP1A\|USP33\|SLC38A2\|IMPACT\|RRAGC\|MLST8\|SEH1L |
| **Cellular response to reactive oxygen species** | #N/A | ABL1\|RHOB\|ECT2\|HSF1\|MAPT\|PEX14\|PEX5\|RPS3\|SQSTM1\|CCS\|SETX |
| **Endocytic recycling** | #N/A | ATP6AP1\|EIPR1\|VPS26A\|SNX17\|STX6\|EHD4\|EHD2\|VPS29\|CCDC93 |
| **Execution phase of apoptosis** | #N/A | BNIP1\|CASP2\|CASP7\|CASP8\|DNASE2\|STK24 |
| **Negative regulation of DNA metabolic process** | #N/A | ABL1\|DACH1\|H1-4\|HSF1\|EXOSC10\|RPS3\|TIMELESS\|SUB1\|PDS5A\|GTPBP4\|DNAJC2\|GNL3L\|TIPIN\|CAMSAP3 |

#N/A: no DEPs were identified of quantified on this pathway.


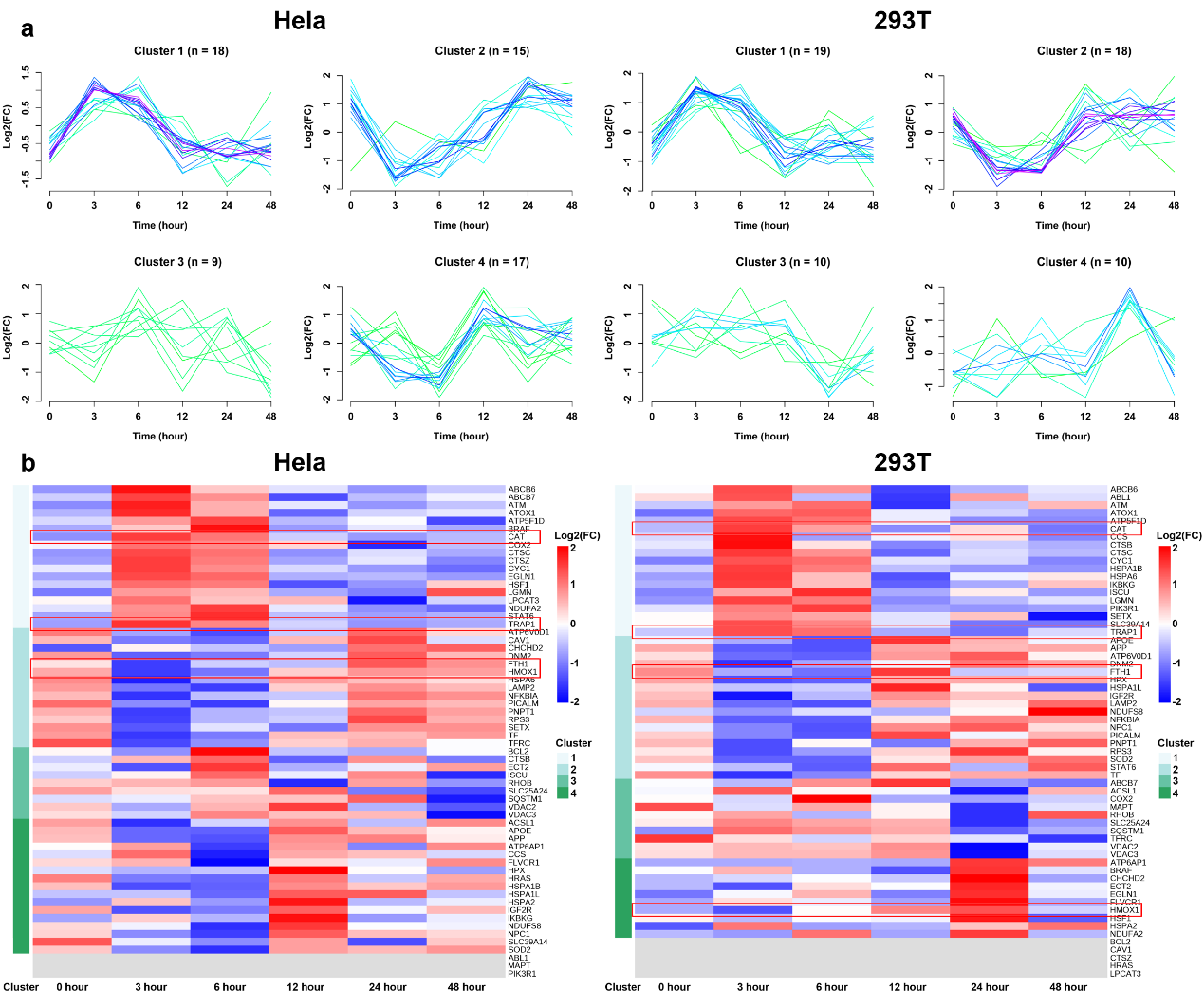


**Figure S2.** The heat maps and clusters of the 62 DEPs involved in oxidative stress, iron ion homeostasis, apoptosis and ferroptosis signaling pathways. a: clusters of the 62 DEPs in Hela and 293T cells, only proteins with quantification data were shown. b: heat maps of the 62 DEPs in Hela and 293T cells.
